# 3D-printed artificial bone scaffolds: the design of materials, the incorporation of bioactive substances, and the integration of vascularized tissue flaps

**DOI:** 10.3389/fbioe.2025.1614727

**Published:** 2025-09-04

**Authors:** Qida Duan, Hongyun Shao, Ning Luo, Fuyang Wang, Liangliang Cheng, Jiawei Ying, Dewei Zhao

**Affiliations:** Department of Orthopedics, Affiliated Zhongshan Hospital of Dalian University, Dalian, China

**Keywords:** biomaterials, microsurgery, 3D-printed, artificial bone scaffold, vascularized tissue flap

## Abstract

With the advancements in tissue engineering, materials science, microsurgery, and the maturation of 3D printing technology, 3D-printed artificial bone scaffolds have provided an innovative strategy that integrates structural bionics and functional synergy for the treatment of large-segment bone defects. Compared with conventional bone grafting, this technology not only precisely reconstructs anatomical geometry and promotes cell migration through porous design, but also, via surface modification, enables accurate loading and controlled release of multiple bioactive factors, thereby actively regulating osteogenesis and angiogenesis, enhancing regeneration efficiency, and overcoming the traditional scaffold limitation of “mechanical support only, lack of biological guidance.” Nevertheless, repair of large-segment defects still faces challenges such as early ischemia, restricted nutrient diffusion, and slow callus formation. To address this bottleneck, the present study summarizes a “vascularization-osteogenesis integration” scaffold design paradigm that combines 3D printing with vascularized bone substitutes, realizing a “scaffold plus vascular-pedicled flap” co-implantation strategy; the vascular network of the flap traverses the entire scaffold, establishing a co-culture microenvironment of endothelial cells and mesenchymal stem cells and maximizing osteogenic and angiogenic efficiency. This review systematically analyzes the biomaterial properties of various 3D-printed bone scaffolds, strategies for loading bioactive factors, and cutting-edge progress in pedicled flap transplantation for bone and vessel regeneration, highlighting their distinctive advantages in vascularization and bioactivity modulation over traditional bone grafting, aiming to promote a paradigm shift from “structural replacement” to “biological function reconstruction” and provide both theoretical innovation and practical guidance for accelerating clinical translation of bone tissue engineering.

## Graphical Abstract

**Figure:**
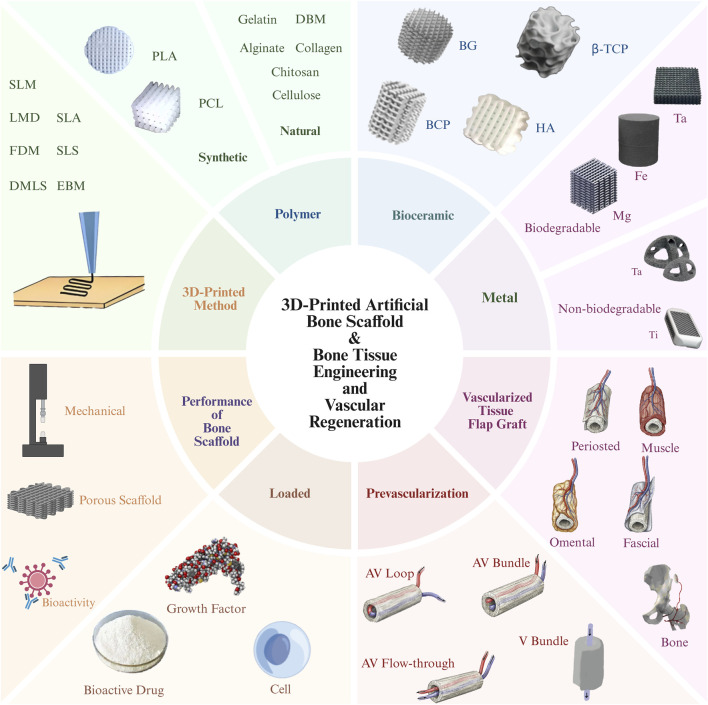
3D-printed artificial bone scaffolds for bone tissue engineering and vascular regeneration: materials, properties and vascularization strategies. Figure created with BioRender.com.

## 1 Introduction

Large-segment bone defects caused by infections, trauma, and malignant tumors are common orthopedic conditions in clinical practice; due to prolonged treatment duration and significant damage to the body, they remain one of the major challenges in current clinical practice. Autologous bone grafting is the gold standard for the clinical treatment of bone defects (Shuck et al., 2023). Owing to its intrinsic osteoconductivity, osteoinductive factors, and absence of immunologic rejection, it yields excellent outcomes in small-to-moderate defects. Nonetheless, limited donor availability, donor-site morbidity, and suboptimal mechanical matching severely restrict its use for large-segment defects (Zhu et al., 2017). Conventional alternatives such as Ilizarov bone transport exploit the tension–stress effect to elicit osteogenesis, yet protracted treatment durations (12–18 months) and high pin-track infection rates (>30%) are notable drawbacks (Malkova and Borzunov, 2021); although the Masquelet induced-membrane technique restores local function more rapidly, its high cost, requirement for a second surgery, and substantial graft demand likewise fall short of ideal repair for extensive defects. These limitations drive ongoing clinical efforts to pursue superior solutions.

In recent years, rapid advances in biomaterials and tissue-engineering technologies have offered new avenues, with 3D printing—owing to its high precision and customizability—showing great promise for fabricating biomimetic scaffolds to mend large-segment defects (Arif et al., 2023). Efficient bone regeneration, however, relies heavily on dynamic crosstalk within the “vascular–osteogenic niche,” where endothelial cells secrete growth factors such as PDGF-BB and VEGF to initiate osteogenic differentiation, and nascent bone matrix releases chemokines such as SDF-1α to guide directional vessel ingrowth (Menger et al., 2022). This interdependence stems from the fact that bone cells require an ample blood supply for nutrient support (Wang et al., 2025a). During reconstruction of large defects, scaffold-resident cells are often distant from host vasculature and thus deprived of adequate perfusion (nutrients and oxygen), resulting in apoptosis rates exceeding 60% and constituting a pivotal barrier to successful healing (Rouwkema and Khademhosseini, 2016).

To overcome this hurdle, investigators are propelling vascularized bone scaffolds forward via synergistic innovations in bioactive design, pre-vascularization techniques, and surgical flap integration. In the realm of bioactive design, Wang R. et al. (2024) fabricated a 3D layered composite scaffold (DFO@GMs-pDA/PCL-HNTs, DGPN) incorporating a deferoxamine (DFO) delivery system; gelatin microspheres preserved DFO activity for 7-day sustained release, activated the HIF-1α pathway to spur angiogenesis, and, together with 1 wt% halloysite nanotubes, synergistically enhanced osteogenic differentiation and matrix mineralization, validating a “coupled angiogenic–osteogenic regeneration” concept in a rat calvarial defect model. Zhou et al. (2025) devised a 3D hollow tubular H-BCP@SDF-1α/EPC composite scaffold that recruited mesenchymal stem cells via SDF-1α and delivered endothelial progenitor cells, markedly accelerating synchronized bone–vessel regeneration in a rabbit steroid-induced femoral head necrosis model. Regarding pre-vascularization, advances in microsurgery have diversified methods for constructing functional microcirculation *ex vivo*; using a bioreactor approach, Huang J. et al. (2022) pre-vascularized scaffolds to enhance intragraft vessel formation Prevascularization refers to the process of establishing a functional microvascular network in tissue or within a scaffold via tissue engineering methods prior to transplantation or implantation (Rouwkema and Khademhosseini, 2016). At present, there are various approaches to vascularizing artificial bone scaffolds, including the implantation of an arteriovenous loop (AVL), an arteriovenous bundle (AVB), an arteriovenous flow-through (AVFT), or a venous bundle (VB) within the scaffold, as well as placing the graft in a muscle pouch (MP); all of these methods can accelerate bone and vessel ingrowth around the scaffold (Beier et al., 2010; Wang et al., 2010)From a surgical integration standpoint, directly grafting a pedicled vascularized flap onto a 3D-printed scaffold not only boosts its angiogenic capacity—circumventing the time required for *ex vivo* pre-vascularization—but also rapidly re-establishes perfusion at the defect site, effectively promoting intra-scaffold bone ingrowth and defect repair. Together, these synergistic approaches represent cutting-edge avenues for surmounting the vascularization bottleneck in large-segment bone regeneration (Simunovic and Finkenzeller, 2021).

A comparison of autologous grafting and novel scaffold technologies in clinical practice (Table 1) reveals marked contrasts in advantages and limitations. Although autografts offer superb biocompatibility, they are constrained by donor-site availability and complication risks; conversely, innovative scaffold systems—leveraging advanced materials (e.g., bio-ceramic composites, magnetic hydrogels) and vascularization tactics—circumvent size limitations in large defects and enable functional customization such as antibacterial activity and controlled drug release. Nevertheless, thorough investigation is still required into long-term degradation kinetics relative to bone regeneration rates, as well as precise modulation of the local immune microenvironment, to expedite clinical translation of these novel scaffolds.

**TABLE 1:** Comparison between autologous bone grafts and novel 3D-printed scaffolds.

Criteria	Autologous bone graft	Novel 3D-Printed scaffold	References
Biocompatibility	No immune rejection (optimal)	Material-dependent(e.g., PCL/HA composites mimic natural bone)	Sun et al. (2025)
Osteoinductivity	High (contains native growth factors)	Requires exogenous factors (e.g., BMP-2/VEGF loading)	Chen et al. (2020a)
Donor limitation	Severe (high risk of donor site complications)	None (mass-producible)	Wang et al. (2024a)
Angiogenic capacity	Host-dependent vascular ingrowth (slow)	Enhanced by prevascularization/growth factor release (e.g., DFO sustained-release systems)	Zhou et al. (2025)
Mechanical strength	Matches host bone	Tunable (e.g., PLGA/nHA/GO comparable to trabecular bone)	Tong et al. (2025)
Clinical maturity	Established (gold standard)	Partially in clinical trials	Shuck et al. (2023)

## 2 3D-printed methods

Also known as additive manufacturing (AM), 3D-printed is an emerging rapid prototyping technique in recent years that builds objects layer by layer from digital model files using various materials (Yu H. et al., 2024). It allows one-step fabrication without molds, eliminating the cumbersome stages of conventional manufacturing; beyond enabling complex geometries unattainable by traditional processes, it affords high dimensional accuracy, strong reproducibility, and facile scalability. As summarized in Table 2, variations in energy source and feedstock form have spawned distinct AM modalities, including selective laser sintering (SLS), selective laser melting (SLM), direct metal laser sintering (DMLS), laser metal deposition (LMD), stereolithography (SLA), electron beam melting (EBM), and fused deposition modeling (FDM), each with unique application niches and technical merits.

**TABLE 2:** Comparison of 3D-printed technologies in bone scaffold manufacturing.

Technology	Suitable for metal printing	Energy source	Powder melting mechanism	Printing environment	Materials used	Mechanical Properties	Biocompatibility	Vascularization potential	Typical Applications	References
SLS	No	Laser	Partial sintering, particle fusion	Inert gas	Polymers, bioceramics	Moderate strength (non-load-bearing)	Good (optimized by polymer-bioceramic composites)	Porous structure enhances cell infiltration	Customized bone tissue scaffolds	Li et al. (2025)
SLM	Yes	Laser	Full melting, liquid pool formation	Inert gas	Metal	High strength (load-bearing)	Excellent (orthopedic implants)	Enhanced via pore optimization	Load-bearing implants (hip joints, spinal cages)	Yan et al. (2025)
DMLS	Yes	Laser	Local melting, particle fusion	Inert gas	Metal	High strength (slightly lower than SLM)	Excellent (bone implants)	Pore-controlled vascularization	Dental implants	Hwang et al. (2022)
LMD	Yes	Laser	Powder/wire melting and deposition	Inert/Open	Metal	High strength, gradient properties	Good (functional modifications possible)	Supports directed vascularization	Surface-modified bone implants	Azhar et al. (2021)
EBM	Yes	Electron beam	Full melting (vacuum)	Vacuum	Metal	High strength (load-bearing)	Excellent (osseointegration)	Promotes endothelialization	Large bone defect repair (pelvis)	Latif and Garg (2020)
SLA	No	UV light	Photopolymerization	Open	Photosensitive resins, biohydrogels	Low-moderate (soft tissue)	Superior (biohydrogels)	Supports microvascular networks	Cartilage repair, craniofacial scaffolds	Jeon et al. (2025)
FDM	No	Heated nozzle	Thermoplastic extrusion	Open	Polymers, bioactive glass	Low-moderate (small bone defects)	Good (biodegradable scaffolds)	Supports bone tissue engineering	Periodontal scaffolds, low-cost prototypes	Zotti et al. (2025)

In fused deposition modeling (FDM), a heated nozzle melts thermoplastic feedstocks—such as polylactic acid (PLA), polycaprolactone (PCL), or PCL–hydroxyapatite composites—into a semi-molten filament that is deposited layer by layer onto a build platform to create a three-dimensional construct (Wickramasinghe et al., 2020). The method features low printing cost, a wide build volume, and broad material compatibility; nonetheless, intrinsic drawbacks persist: (i) limited resolution, with a typical layer thickness of ∼0.4 mm producing pronounced stair-stepping; (ii) inadequate mechanics, as pure PCL scaffolds exhibit compressive strengths of only 8–10 MPa; and (iii) constrained structural complexity, since overhangs require additional supports, impeding fabrication of highly intricate geometries. In recent years, material-modification strategies have markedly enhanced the performance of FDM-printed scaffolds (Sun et al., 2025). For example, Salehi et al. (2023) incorporated Baghdadite nanoparticles into a PLA matrix, boosting scaffold compressive strength by 40% and elevating the elastic modulus to 50–200 MPa (approaching cancellous bone), while subsequent loading with vascular endothelial growth factor (VEGF) endowed concurrent osteogenic and angiogenic functionality.

Selective laser sintering (SLS) employs a high-energy laser to selectively fuse polymer powders such as PLA or PCL, layer-wise producing porous scaffolds with interconnected channels. Compared with FDM, SLS offers three principal merits: (i) high-resolution fabrication, with layer heights of 0.1–0.2 mm and achievable porosities of 60%–80%; (ii) excellent interlayer fusion, yielding shear strengths >15 MPa; and (iii) the capacity to print complex biomimetic architectures without supports, including trabecular-like pores that facilitate cell infiltration. These attributes make SLS an ideal choice for repairing non-load-bearing bone defects such as those in the calvaria or mandible (Liu et al., 2020; Doyle et al., 2015). To further enhance the performance of SLS-fabricated scaffolds, Meng et al. (2020) developed an SLS post-processing technique involving NaCl particle embedding. Through an *in-situ* remelting–re-solidification mechanism, the method markedly increased the microstructural density and mechanical strength of PCL scaffolds. The treated scaffolds exhibited a compressive modulus of 3027.8 ± 204.2 kPa and a compressive strength of 208.8 ± 14.5 kPa, representing 2.1-fold and 1.8-fold increases over untreated controls. After 24 weeks *in vivo*, the scaffold retained structural integrity and mechanical stability, with a 15% lower degradation rate than untreated samples; newly formed tissue infiltrated the porous regions well, confirming favorable mechanics-to-degradation matching. Critically, the post-processing significantly delayed mechanical deterioration.

Metal AM techniques—including SLM, EBM, DMLS, and LMD—employ high-energy laser or electron beams to process metal powders, yet differ in melting mechanism: SLM, EBM, and LMD fully melt the powder, whereas DMLS effects only partial sintering. Notably, EBM is unique in being solely applicable to metals and requiring operation under vacuum. Both SLM and EBM can fabricate high-strength alloy implants with compressive strengths >500 MPa—suitable for load-bearing sites such as hip prostheses or spinal cages—and allow the design of 20%–80% biomimetic gradient porosities that substantially enhance osseointegration (Zhao et al., 2025). Moreover, SLM shows formidable capability for intricate geometries; for instance, Wen et al. (Revilla-León et al., 2022) successfully produced cardiovascular porous stents 2–5 mm in diameter with 200–500 μm pores, underscoring its precision complexity. Nevertheless, these high-energy beam processes face intrinsic challenges: rapid melt-solidification induces residual stresses that can spawn microcracks. Studies indicate that such effects can reduce the fatigue strength of DMLS-fabricated scaffolds by roughly 15% (Revilla-León et al., 2022). Additionally, the printed scaffolds often possess high surface roughness, which is unfavorable for cell adhesion.

Stereolithography (SLA) dates back to 1981, when (Kodama, 1981) first used ultraviolet light to selectively cure liquid resin into a 3-D solid, laying the foundation for photopolymer-based 3D printing. Conventional SLA uses UV lasers with galvanometer mirrors to precisely steer the beam, layer-by-layer curing photopolymer slurry to create ultra-high-resolution scaffolds (≈25 μm layers, Ra ≈ 3–6 μm), conferring unique advantages for delicate anatomical reconstructions such as auricular cartilage regeneration or cranio-maxillofacial repair (Gao et al., 2024; Slavin et al., 2023). However, SLA faces serious biosafety concerns: the process relies on acrylate reactive diluents (e.g., HDDA, TEGDMA) for viscosity control, and residual monomers elicit notable cytotoxicity (extract cell viability <70%). To address this bottleneck, (Melchels et al., 2009), pioneered a nontoxic-diluent SLA approach. They designed star-shaped poly(D,L-lactide) (PDLLA) macromers, used methacryloyl chloride end-capping for photopolymer cross-linking, and employed ethyl lactate as a green diluent, thereby fabricating porous PDLLA scaffolds. Compared with conventional SLA scaffolds containing reactive diluents, this strategy achieved notable improvements: (i) compressive modulus increased from 80 ± 10 MPa to 120 ± 15 MPa; and (ii) extract cytoviability rose from <70% to >95%. This synergistic “star-topology cross-linking/green diluent” strategy not only endows the scaffold with favorable porosity for vascular infiltration and directed osteoblast migration but also offers a breakthrough platform for dual mechanical-and-bioactive regeneration in cranio-maxillofacial defects.3D-printing technology enables the precise fabrication of bone scaffolds that closely match human anatomy. Such three-dimensional porosity furnishes an ideal microenvironment for osteoblast adhesion, proliferation, and functionality while delivering essential mechanical support to the defect site (Szczepańczyk et al., 2021). It is noteworthy that a scaffold’s mechanical performance dictates its load-bearing capacity, whereas its chemical composition profoundly influences the angiogenic potential at the implantation site (Sleem et al., 2025). Therefore, material selection is a pivotal determinant of effective bone regeneration and remains a central focus and frontier hotspot in bone tissue engineering research.

## 3 Materials of 3D-printed artificial bone scaffolds

By integrating 3D-printed with diverse biomaterial scaffolds, adequate space and mechanical support are provided for bone repair, enabling both bone and blood vessels to absorb nutrients, exchange gases, and eliminate waste within the scaffold’s three-dimensional structure. An ideal 3D-printed artificial bone scaffold material should meet these requirements: high-precision single-step fabrication of a three-dimensional porous structure with sufficient mechanical strength, excellent biocompatibility, and a strong material–cell interface (Barabaschi et al., 2015). We provide an overview of the multiple materials—polymers, bioceramics, and metals—used in 3D-printed bone scaffolds, highlighting how their application has substantially advanced bone tissue engineering.

### 3.1 Polymers

Polymers for 3D-printed are generally divided into two groups: natural polymeric materials and synthetic polymeric materials. Synthetic polymers are produced under controlled conditions, giving them adjustable mechanical properties, crosslinking, porosity, elastic modulus, and degradability (Amiryaghoubi and Jahanban Esfahlan, 2024). In contrast, natural polymers have a higher degradation rate, lower mechanical strength, better cell-binding capacity, and improved biocompatibility, low irritability, and favorable water absorption and permeability (Osman et al., 2022) Some natural polymers also possess immunological properties, making them suitable for 3D-printed bone scaffolds (Kobbe et al., 2020).

**FIGURE 1:**
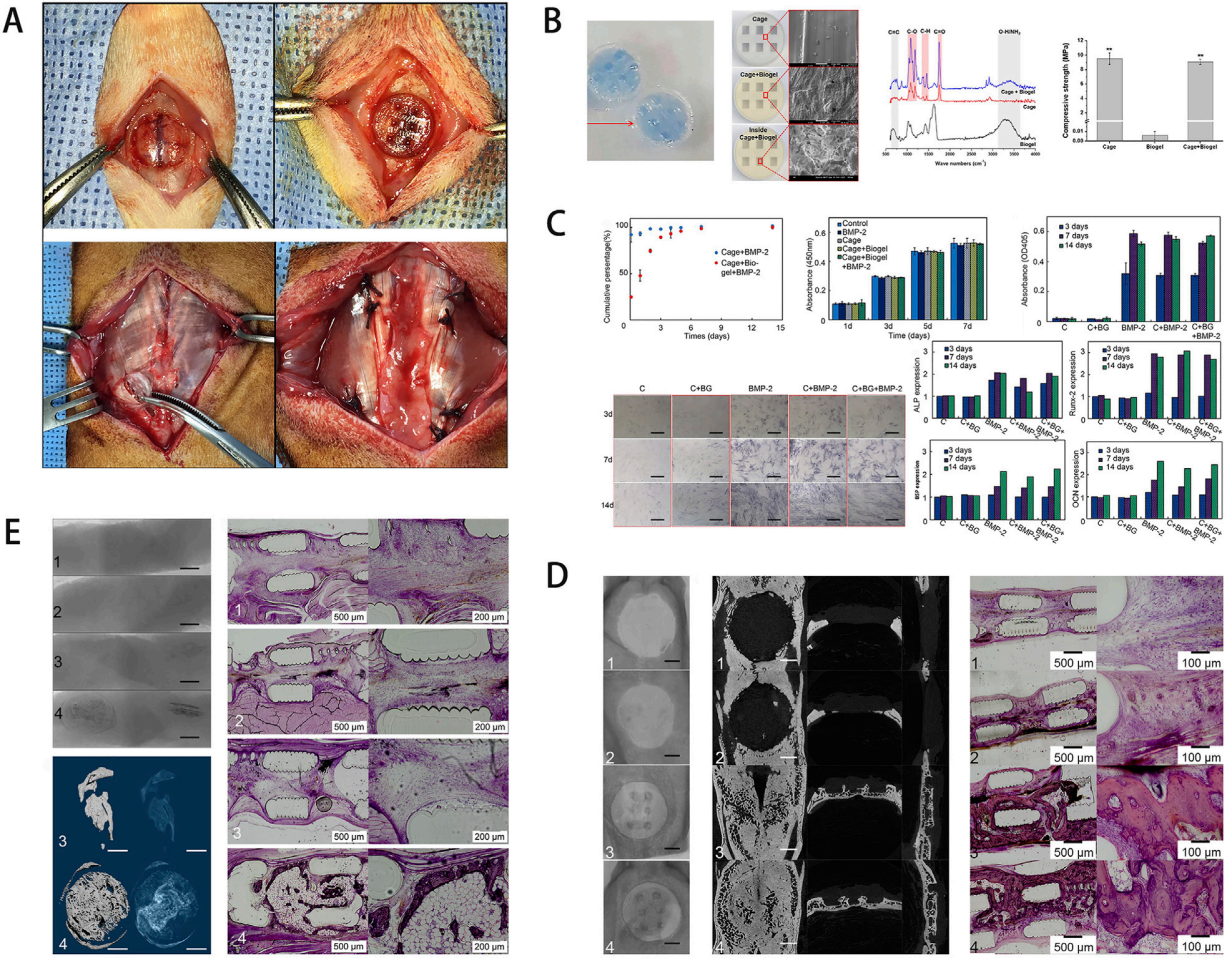
**(A)** Schematic of the rat calvarial defect and ectopic ossification models. **(B)** Scaffold characterization via staining, SEM, FT-IR analysis, and mechanical testing of the hydrogel-coated scaffold. **(C)** Assessment of growth factor release, cell viability, and osteogenic gene expression. **(D)** *In vivo* results, including micro-CT and histological staining. **(E)** Ectopic bone formation showing scaffold-induced osteogenesis in muscle tissue, with the experimental group exhibiting more organized bone formation. [Figure adapted from Cha et al. (2021)].

Among synthetic polymers, PLA is favored for its excellent biocompatibility, biodegradability, and processability (Jee et al., 2024). Studies highlight the critical role of architectural design: Cavo and Scaglione (2016) fabricated collagen-coated PLA scaffolds with pores of 300, 600, and 900 μm, and found that the 600 μm group exhibited the highest compressive strength, cell proliferation, and adhesion *in vitro*. Cha et al. (2021) loaded bone morphogenetic protein-2 (BMP-2) and Biogel into a PLA cage scaffold and, after creating an 8 mm circular defect in rat calvaria, implanted the scaffold to repair the defect. Additionally, four muscle pouches (10 mm in length and depth) were created bilaterally in the latissimus dorsi of each rat. Scaffolds were inserted into these pouches and sutured closed for ectopic ossification experiments. At 2 weeks, minor bone formation was observed in the defect area, and ALP activity and BV/TV ratio were higher than in the scaffold-only control. By 8 weeks, extensive bone formation with neovascularization was evident (Figure 1). Moreover, PLA’s degradation period extends to 24 weeks, offering prolonged mechanical support and protection for the regenerating tissue. Common natural polymers in artificial bone scaffolds include demineralized bone matrix (DBM), a variety of proteins, and chitosan. Hogan et al. (2023) combined photo-reactive gelatin methacrylate nanoparticles (GNP-MAs) with DBM to develop a 3D-printed GNP-DBM-NP scaffold. The scaffold’s collagen content (217 ± 8 μg/mg) greatly exceeded that of native bone (11.4 ± 4.5 μg/mg) and markedly upregulated osteogenic markers RUNX2 and OCN, demonstrating its potential as a cell carrier. However, the DBM manufacturing process can vary greatly: complete demineralization reduces mechanical strength and removes critical calcium needed for osteogenesis, whereas insufficient demineralization prolongs degradation and hinders bone ingrowth. These challenges limit its clinical utility. In contrast, synthetic polymeric materials offer excellent mechanical properties and chemical stability, including outstanding heat and corrosion resistance.

### 3.2 Bioceramic

Bioceramic materials, due to their unique chemical composition, are critical for the integration of bone and soft tissues (Brochu et al., 2024).3D-printed bioceramic scaffolds include hydroxyapatite (HA), β-tricalcium phosphate (β-TCP), calcium phosphate cement (CPC), bioactive glass (BG), and newer generations of silicon-based ceramics. These materials exhibit a high affinity for bone and generate a mildly alkaline environment during surface degradation, thereby promoting osteoblast adhesion, proliferation, and bone formation (Ansari et al., 2022). Vallet-Regí et al. (2011) investigated a next-generation porous silica-based bioceramic material for 3D-printed bone scaffolds, loading osteoinductive factors (peptides, hormones, and growth factors) into the porous scaffold to draw bone cells and thereby promote bone repair and angiogenesis. HA accounts for 65% of total human bone mass and is vital for bone growth and preventing bone dissolution (Oonishi, 1991). Hogan et al. (2023) combined photo-reactive gelatin methacrylate nanoparticles (GNP-MAs) with DBM to develop a 3D-printed GNP-DBM-NP scaffold. The scaffold’s collagen content (217 ± 8 μg/mg) greatly exceeded that of native bone (11.4 ± 4.5 μg/mg) and markedly upregulated osteogenic markers RUNX2 and OCN, demonstrating its potential as a cell carrier. In exploring HA development, Cox et al. Hench (1989) prepared bone scaffolds via 3D-printed using a composite powder of HA and polyvinyl alcohol (PVOH). The flow properties of the HA-PVOH mixture affected the scaffold’s mechanical properties, microstructure, and porosity. The resulting scaffold had a compressive strength of 0.88 ± 0.02 MPa, and radiographic plus histological examinations demonstrated its critical role in supporting bone conduction.

**FIGURE 2:**
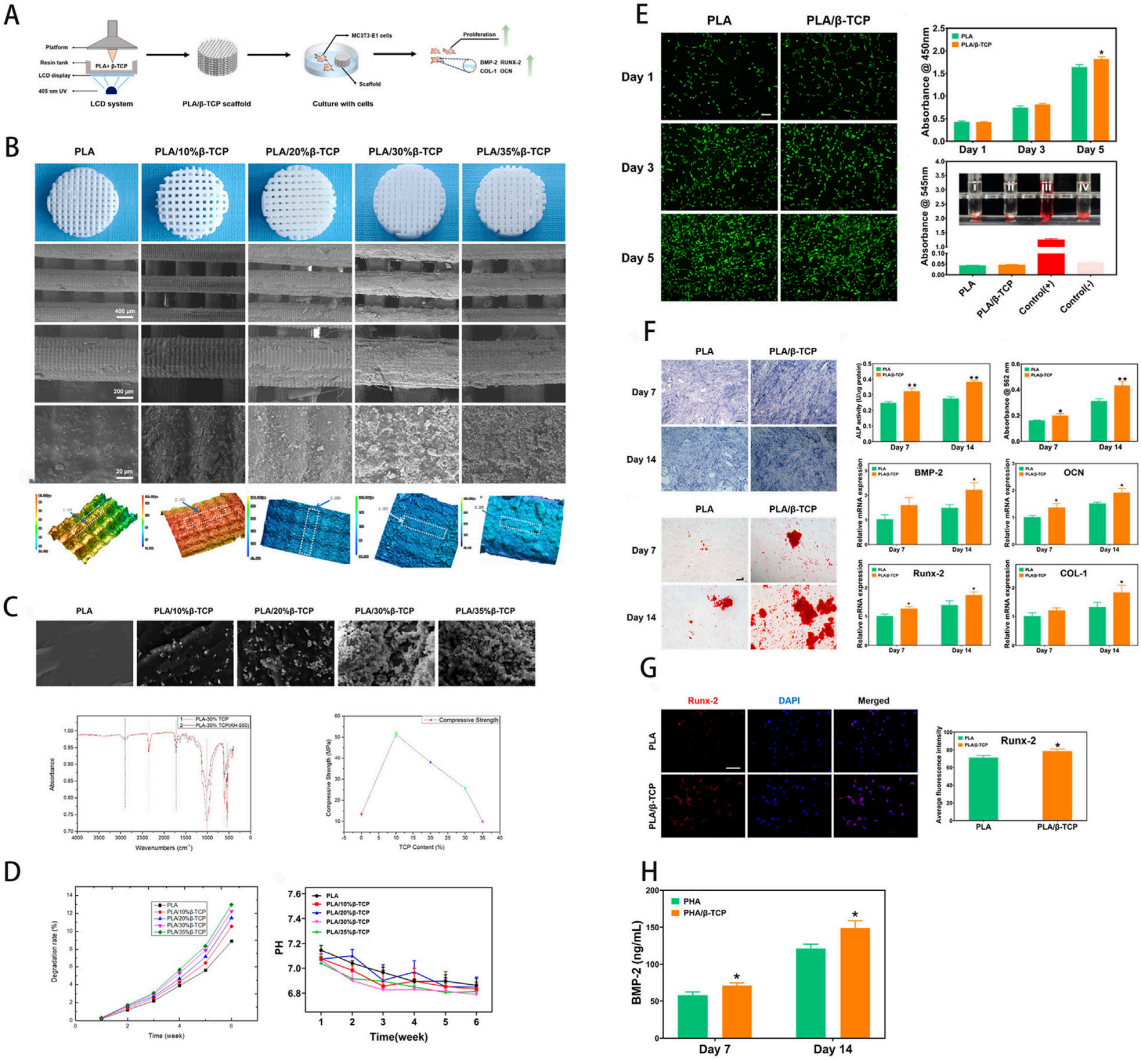
**(A)** Schematic of scaffold fabrication via LCD-based UV-assisted layer-by-layer curing of PLA/β-TCP composite resin. **(B)** Macroscopic and SEM imaging of the scaffold, with surface roughness analysis. **(C)** Compressive fracture morphology, FTIR spectra, and comparative compressive strength analysis. **(D)** *In vitro* degradation profile, assessing weight loss and pH variations in simulated body fluid. **(E)** Osteoblast proliferation (fluorescence imaging), CCK-8 cytocompatibility, and hemolysis assays. **(F)** Osteogenic differentiation potential and gene expression analysis. **(G)** Quantitative immunofluorescence analysis of osteogenesis. **(H)** Growth factor expression induced by the scaffold. [Figure adapted from Wang B. et al. (2024)].

Phosphates are frequently employed in bone-tissue scaffolds owing to their excellent biocompatibility. Among them, β-TCP and CPC are widely applied in artificial bone scaffolds. These materials can be 3D-printed into scaffolds with pore sizes from 5 μm to 500 μm; their intrinsic osteoinductivity combined with trabecular-like porosity enhances bone-healing capacity (Bohner et al., 2020). Wang B. et al. (2024) evaluated PLA/β-TCP composites made by LCD-based 3D printing, fabricating scaffolds with β-TCP (0%, 10%, 20%, 30%, 35%); increased loading enriched surface particles, and the 10% formulation achieved 52.1 MPa compressive strength while markedly enhancing MC3T3-E1 proliferation and osteogenic differentiation (Figure 2). This study underscores the potential of 3D-printed composite scaffolds to optimize surface bioactivity and enhance bone repair, providing key insights for coupling with vascularization strategies to treat bone defects. Gbureck et al. (2007) deviated from convention by reacting biphasic α/β-TCP powders with phosphoric acid during 3D printing, forming CPC scaffolds with programmable architectures *in situ* at low temperature. The resulting scaffolds exhibited initial compressive strengths of 0.9–8.7 MPa, which rose to 22 MPa after 3 min phosphoric acid treatment; hydrothermal conversion increased porosity by 13% and reduced strength to 15 MPa (still above native bone), while markedly accelerating *in vivo* resorption. CPC is widely used in orthopaedic implants for its superior bioactivity, yet challenges remain concerning stiffness requirements and control over degradation rate (Zhao C. et al., 2022). Bioactive glass (BG), a silicate-based material, contains calcium, phosphorus, and network-modifying oxides such as CaO and MgO. Its highly reactive surface promotes amorphous calcium phosphate formation, facilitating protein adsorption and cell attachment (Chen et al., 2024). Such properties enable BG to bond readily with bone matrix, conferring strong osteoconductivity (Ribas et al., 2019). Ions released during BG degradation (Na^+^, Ca^2+^, SiO_4_^4-^, PO_4_^3-^) can stimulate osteogenesis and angiogenesis (Peric Kacarevic et al., 2020). The degradation rate of BG can be tuned via its composition, offering better support for endogenous bone remodeling (Kaou et al., 2023). However, like most bioceramics, the inherent brittleness of BG limits its mechanical performance, rendering it unsuitable for load-bearing sites (Baino et al., 2019).

### 3.3 Metals

During the early stages of bone defect repair, the artificial bone scaffold must bear the entire mechanical load. Based on the mechanical loads of different anatomical sites, the scaffold’s structural integrity must be maintained for at least 3–12 weeks (upper limbs) or 12–24 weeks (lower limbs) to match the bone healing process and avoid premature mechanical failure that could impede regeneration (Zheng et al., 2014). Metallic materials display superior biomechanical properties and are widely used in load-bearing scaffolds for weight-bearing bones. Currently, titanium (Ti) alloys, stainless steel (316L), and cobalt-chromium-molybdenum (Co-Cr-Mo) are combined with 3D-printed to produce porous structures that provide space for cells, tissues, blood vessels, and nerves to grow, thereby promoting bone ingrowth (Bandyopadhyay et al., 2019; Huang G. et al., 2022; Lee et al., 2018).

Ti alloys possess high corrosion resistance and outstanding mechanics; 3D printing enables precise tuning of pore architecture, reducing the stress-shielding ratio from 35% (solid metal) to 8% (porous), which favours bone growth and healing (Li S. et al., 2021). Clinical studies indicate that 3D-printed porous-titanium implants show clear advantages in cranio-maxillofacial surgery: their anatomical fit reduces loosening risk, and 6–24 months follow-up confirms robust osseointegration with low complication rates, achieving 86.7% success in calvarial symmetry restoration and foot-ankle deformity correction (Park et al., 2016; Hamid et al., 2016). Moreover, bi-functional modifications of porous Ti—such as bioactive coatings or antibacterial treatments—further boost bioactivity and infection resistance, opening avenues to couple mechanical stability with biological performance (Cui et al., 2025). Collectively, these advances signal a shift from “structural substitution” to “functional regeneration” in 3D-printed titanium scaffolds. In recent years, porous tantalum (Ta) has garnered extensive attention as a novel scaffold material for bone defect repair (Huang et al., 2021). Porous Ta scaffolds manufactured via selective laser melting (SLM), for applications like spinal vertebral bodies, tibial implants, and acetabular prostheses, have proven effective in various clinical scenarios (Baino et al., 2019). Compared with Ti, SLM-fabricated porous Ta (50%–80% porosity) exhibits an adjustable elastic modulus of 2–3 GPa—much lower than porous Ti (10–30 GPa) and closely matching native bone (trabecular 0.1–0.5 GPa, cortical 12–18 GPa) (Zhao et al., 2024). Compression tests show porous Ta scaffolds yield at 450 ± 30 MPa, and they also outperform porous Ti in fatigue resistance (Liu B. et al., 2022; Jin et al., 2025). These properties markedly reduce stress shielding (<10%) and thus facilitate bone-defect healing (Liu B. et al., 2022). Porous Ta is also highly biocompatible; interconnected pores (300–500 μm) encourage endothelial migration and capillary ingrowth, thereby amplifying osteogenesis (Jin et al., 2025; Wu MH. et al., 2022).

Biodegradable metals such as Mg, Fe and Zn have likewise been 3D-printed into bone scaffolds, offering promising therapeutic outcomes for defect repair. Fe-based alloys possess adequate strength for skeletal implants, yet their *in vivo* corrosion must be accelerated to match bone-healing kinetics (Li et al., 2019; Mishra and Pandey, 2020). Using inkjet 3D printing, Chou et al. (2013) fabricated degradable Fe-Mn scaffolds (36.3% porosity) with 350 ± 20 MPa compressive strength and 100–120 GPa modulus; *in vitro* studies confirmed osteoblast compatibility, while Mn doping raised *in vivo* corrosion from 0.05 to 0.2 mm yr^-1^—closer to the bone-formation rate (0.5–1 mm yr^-1^). Shuai et al. (2020) produced SLM-built Fe-35 wt% Mn scaffolds whose tensile strength (144 MPa) and modulus (53.3 GPa) approximate cortical bone, while exhibiting a high degradation rate (0.23–0.306 mm yr^-1^) in simulated body fluid. After 28 days’ degradation, the modulus and yield strength of Fe-35 wt% Mn scaffolds resemble those of trabecular bone.

Compared with Fe, Mg degrades faster and is less strong, yet it offers good biocompatibility, low thrombogenicity and intrinsic osteo-inductivity (Hermawan, 2018; Ibrahim et al., 2017). Zhang et al. (2020) designed intricate porous dental implants from resorbable Mg alloys; finite-element analysis showed no stress concentration and satisfactory mechanical performance. The porous design facilitates stress transfer through cancellous bone, enhancing load sharing. Cell assays revealed that Mg^2+^ diffusion stimulates osteoblast proliferation; at 0.25 mM d^-1^, Mg^2+^ via TRPM7 upregulated hBMSC osteogenesis (Runx2 mRNA × 2.5), but concentrations >0.5 mM d^-1^ induced apoptosis (viability <70%) (Putra et al., 2020; Karunakaran et al., 2020). Zhang et al. (2022) fabricated porous Mg-particle scaffolds coated with dicalcium phosphate dihydrate (DCPD) via 3D printing. The DCPD-coated Mg scaffold exhibited a compressive strength of 5.38 ± 0.87 MPa, adequate for cancellous-bone support. Its porosity (57.6% ± 3.9%) closely mirrors that of cancellous bone. A further advantage is its larger pore diameter relative to trabecular bone, which favours neovascularisation and osteogenesis.

Zinc, whose degradation rate lies between Fe and Mg, shows promise for bone implants (Li et al., 2020). Fully resorbable Zn^2+^ serves as a cofactor for >300 enzymes, is vital to immune and neural development, and exhibits anti-resorptive and antimicrobial effects; mechanically, Zn alloys resemble human bone more closely than Mg or Fe alloys (Jiang et al., 2022). Additive manufacturing can yield Zn lattices with trabecula-like modulus, minimising stress shielding (Wang et al., 2020). However, Zn’s low melting point, high vapour pressure and powder-processing issues complicate 3D printing of Zn scaffolds (Zhou et al., 2022). Yang M. et al. (2022) reinforced Zn implants with carbon nanofibres (CNF) and applied a La coating to improve interfacial compatibility. La forms strong coordinate covalent bonds with oxygenated groups on CNF and alloys with the Zn matrix, heightening metallurgical bonding. This modification raised the composite’s tensile strength from 180.2 MPa to 243.4 MPa. The alloy accelerated radial-bone defect healing in rabbits, outperforming pure-Ti scaffolds. Nevertheless, its elongation (27.82% ± 18.35%) is lower than that of binary Zn-Mn alloys (83.96% ± 2.36%) (Jiang et al., 2022; Zhang et al., 2021). Existing 3D-printed materials have, to some degree, sped up bone defect repair. Over the past few decades, tissue engineering has made notable progress in scaffold design and structural optimization, particularly in using 3D-printed to replicate the biological, mechanical, and chemical properties of target tissues. These advancements help in producing artificial bone scaffolds that are closer to the function and structure of natural bone tissue, thereby minimizing disparities between engineered materials and native tissue, and ensuring effectiveness in bone repair.

## 4 The performance of 3D-printed artificial bone scaffolds

An ideal artificial bone scaffold should possess the following attributes: good mechanical properties, a porous structure that allows for cell and protein infiltration, and an inherent bioactivity that can enhance bone repair and angiogenesis. It must exhibit sufficient strength to meet the early mechanical demands of the implantation site (Fu et al., 2022). The porous structure of the scaffold, which includes both porosity and pore size, is critical for facilitating cell survival and tissue growth (Huang et al., 2025). The intrinsic bioactivity of the scaffold material also impacts angiogenesis at the implantation site and influences its potential to repair bone defects (Vidal et al., 2020).

### 4.1 Mechanical properties

The mechanical properties of 3D-printed bone scaffolds are critical parameters for evaluating their efficacy and clinical potential, and the mechanics of different materials are likewise influenced by multiple factors. Elastic modulus serves to evaluate scaffold stiffness and deformation under load, and bones of distinct anatomical structures exhibit different moduli. According to (Olszta et al., 2007), cortical bone possesses an elastic modulus of 15–20 GPa, whereas mature trabeculae display values around 1 GPa. Compressive strength is an important indicator of a scaffold’s capacity to resist external loads. In terms of compressive strength, cortical bone ranges from 100 to 200 MPa, whereas trabecular bone lies between 2 and 20 MPa (Petersen et al., 2018). Therefore, scaffold materials should be designed with mechanical properties closely matching those of the host bone. Liu et al. (2009) reported that a 3D-printed borosilicate glass lattice scaffold achieved a compressive strength of ∼10 MPa, a value closer to documented cancellous bone strength. Flexural strength denotes the maximum stress a material can endure under bending load. Scaffold stiffness influences the proliferation and differentiation of osteogenic cells within the scaffold (El-Rashidy et al., 2021). In the study by Murphy et al. (2012), mesenchymal stem cells cultured on collagen–GAG scaffolds of 1.5 kPa stiffness exhibited the highest Runx2 expression, whereas scaffolds of 0.5 kPa promoted SOX9 expression. Fatigue behaviour is likewise crucial; fatigue failure in bone scaffolds arises from cyclic stresses below the ultimate compressive strength that eventually generate cracks (Abdelaziz et al., 2023). As shown in Table 3, increasing porosity lowers compressive strength, elastic modulus, and yield strength. At the same 50% porosity, PCL exhibits a compressive strength of 2–8 MPa, markedly below the 10–80 MPa of PLA. Although tantalum is more biocompatible than titanium alloys, at identical porosity and pore size its compressive strength is only 14–60 MPa compared with 900–1200 MPa for Ti-6Al-4V. However, elastic modulus is not solely governed by porosity; SiO_2_, with a modulus of 10–70 GPa at 50%–80% porosity, far exceeds HA (0.4–0.5 GPa), underscoring intrinsic stiffness. In most cases, material class dictates the upper mechanical limit: metals such as Ti-6Al-4V and ceramic composites exhibit compressive strengths >100 MPa at equal porosity, vastly outperforming polymers and pure bioceramics. Polymer and bioceramic scaffolds typically exhibit lower strength (<50 MPa), yet composite strategies (e.g., PCL/HA) can enhance their performance. We also observe a nonlinear relationship between porosity and strength: within the same material class similar porosities can yield wide strength variations—for example, TCP 2–43 MPa versus HA 9–17 MPa—indicating that pore distribution and microstructure (interconnected versus closed pores) strongly influence performance (Xu et al., 2014; Willems et al., 2014; Slavin et al., 2023).

**TABLE 3:** Characterization of porous structures and mechanical properties in scaffolds composed of various materials.

Material categories	Material Names	Yield Strength (Mpa)	Elastic modulus (GPa)	Pore size (μm)	Porosity (%)	Compressive strength (Mpa)	Degradation time (m)	References
Polymers	DBM	1–5	0.1–0.5	300	—	0.138–0.207	0.5–0.8	Hogan et al. (2023) González González et al. (2022), Popescu et al. (2023), Samantaray et al. (2020), Sun et al. (2022), Sepahi et al. (2021), Lu et al. (2020), Kumar et al. (2019), Zhou et al. (2020), Luo et al. (2017), Sarmast et al. (2024), Miao et al. (2024), Distefano et al. (2023), Yang et al. (2020), Yazdimamaghani et al. (2017), Jiang et al. (2021), Iatecola et al. (2021), Ren et al. (2024), Yu et al. (2024b), Zimmerling et al. (2021), Lu et al. (2012), Ilyas et al. (2022), Qin et al. (2022), Athanasiou et al. (1998), Wang et al. (2019) Mi et al. (2014)
	PLA	52–70	2.6–3.3	200–500	20–70	10–80	6–24	
	PCL	1–10	0.1–0.4	100–500	50–80	2–8	12–36	
	PGA	—	5–10	200–400	50	12–38	1.5–6	
	PLGA	10–50	2–4	—	60–80	1.92–10	<12	
	PEG	1–3	0.1–2	100–500	50–80	1–5	—	
Bioceramics	TCP	2–10	0.18–1	200–1000	30–60	2–43	1–12	
	HA	—	0.4–0.5	—	50–80	9–17	1–12	
	CS	1–3	0.1–0.5	100–500	60	2.31	—	
	BG	0.8–16	0.05–0.08	300–530	65–87	0.42–16.74	<12	
	SiO2	—	10–70	300–500	50–80	20	—	
	SiC	—	—	10–500	40–80	100–250	—	
Metals	Ti-6Al-4V	—	110–117	100–1000	20–60	900–1200	—	
	Ta	—	1.5–3.3	300–800	60–80	14–60	—	
	Mg Alloys	140	41–45	200–500	50–75	—	6–12	
	316L	210–250	190–210	300–600	50–70	80–120	—	
	Co-Cr-Mo	500–600	200–250	—	—	600–1000	—	
	Ni-Ti	300–600	28–83	300–700	50–75	—	—	
Composites	ZrO2/Al2O3	500–900	200–400	100–500	30–50	900–1000	—	
	PCL/HA	2–12	10–30	300–600	50–85	5–12	—	
	PCL/β-TCP	3–10	0.05–0.5	300–500	40–55	5–30	12–36	
	PCL/BG	2–8	0.05–0.15	—	60–85	—	12–36	
	PLA/TCP	2–10	0.1–0.5	300–600	40–70	5–20	—	
	PLA/PGA	—	2–5	—	—	10–40	6–12	
	PLA/SiC	35–50	1–5	200–500	50–70	10–20	12–36	
	HA/PU	1–5	0.03–0.1	—	60–80	3–12	6–24	

### 4.2 Pore geometry, pore size, porosity

The osteointegration efficiency of porous scaffolds is strongly governed by the synergy between their topological attributes (pore shape, pore size, porosity) and the chosen manufacturing process. State-of-the-art 3D-printing platforms—stereolithography, selective laser melting, fused deposition modelling—permit fine control over layer resolution and deposition paths, enabling biomimetic-gradient or complex porous architectures (triangular, helical, hexagonal, trabecular-like pores, etc.) (Dei et al., 2024). Distinct pore morphologies modulate the mechanical micro-environment and biochemical signalling, markedly influencing cell adhesion, proliferation and vascular-network formation. Olivares et al. (2009) modelled spiral and hexagonal lattices at 55% and 70% porosity using finite-element analysis; theoretical evaluation of shear strain and fluid shear stress showed that perfusion-induced shear distribution largely depends on internal pore arrangement. Compared with the hexagonal design, spiral pores afforded superior fluid accessibility, favouring cell attachment. Van Bael et al. (2012) reported that Ti-6Al-4V scaffolds with hexagonal pores most strongly promoted cell proliferation; their many vertices and near-circular outline produced uniform shear fields, minimising mechanical heterogeneity, followed by rectangular pores, whereas triangular pores supported the least growth. Wu F. et al. (2022) implanted cube, gyroid and hexagon bioceramic scaffolds into rabbit latissimus dorsi to compare their *in vivo* angiogenic capacity. Continuous curved channels in gyroid scaffolds guided endothelial cells to migrate along a 0.5–2.1 Pa shear gradient, raising vessel density by 40% over hexagons and elevating VEGF expression 1.8-fold. This benefit correlated with the gyroid’s low permeability, prolonging cell-matrix contact and activating key angiogenic pathways (e.g., PI3K/Akt). Trabecular-mimetic porosity recreates bone’s heterogeneous multiscale network; randomly oriented channels create dynamic flow fields that chemotactically guide mesenchymal-stem-cell migration. Goreninskii et al. (2025) fabricated PEKK scaffolds with trabecular biomimicry and observed MSC adhesion densities of 234 ± 8 cells mm^-2^ versus 121 ± 40 cells mm^-2^ in regular pores.

**FIGURE 3:**
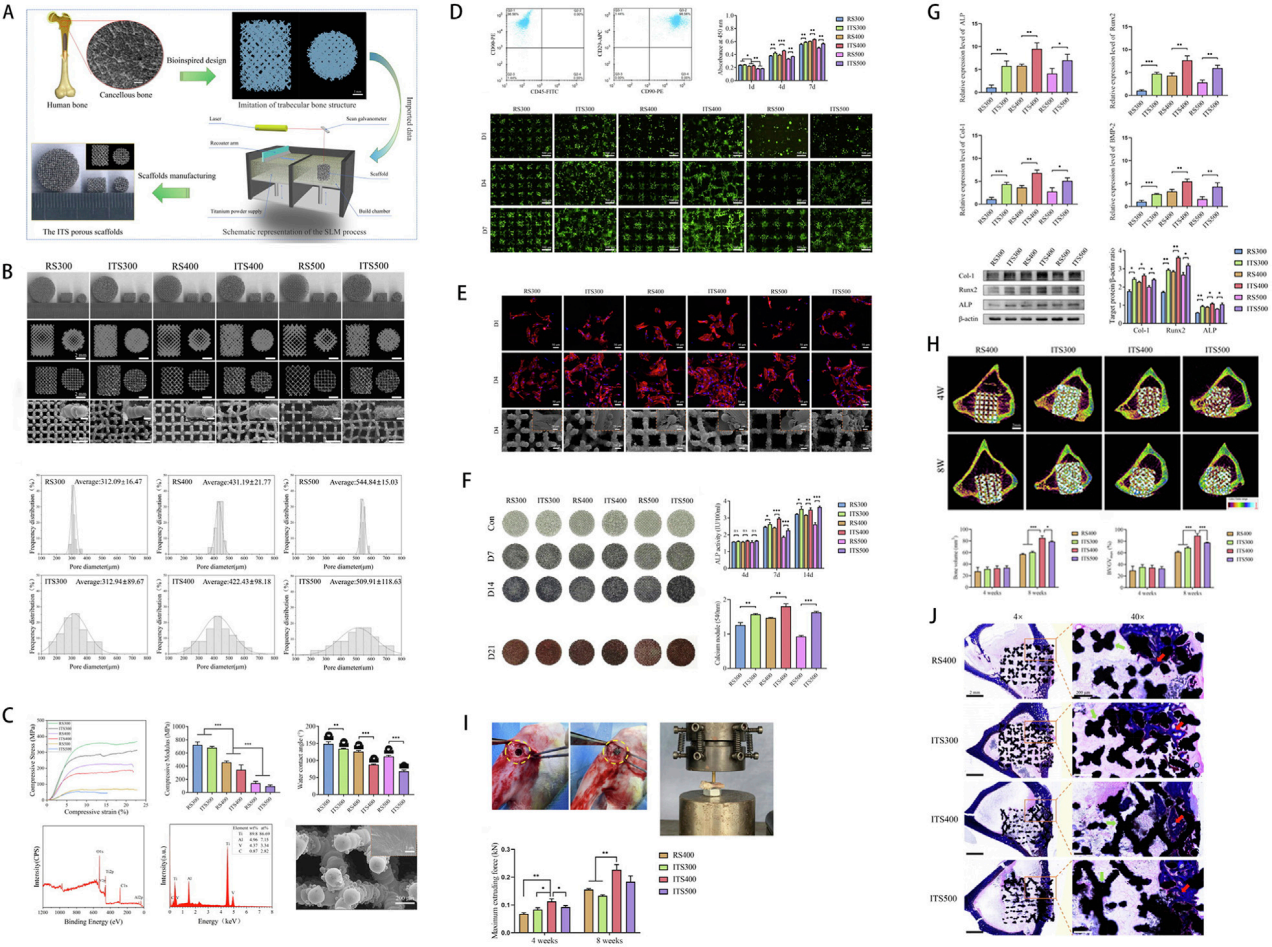
**(A)** Schematic illustration of a Ti6Al4V scaffold with a trabecular bone-mimicking structure fabricated via selective laser melting (SLM). **(B)** Macroscopic and microscopic images of scaffolds from different experimental groups. **(C)** Physicochemical characterization, including stress-strain curves, compressive modulus, wettability, chemical composition, and surface elemental analysis. **(D)** Assessment of osteoblast adhesion and proliferation on the scaffold surface. **(E)** Cell morphology and viability evaluated by fluorescence staining and SEM imaging. **(F)** Osteogenic performance analysis, including osteogenic gene expression, histochemical staining, and quantitative assessment of calcium deposition. **(G)** Expression profiling of osteogenic-related genes and proteins. **(H)** Micro-CT evaluation of bone ingrowth at 4 and 8 weeks post-implantation, with quantitative analysis. **(I)** Push-out testing to assess the interfacial bonding strength between the scaffold and newly formed bone in a rabbit tibial defect model. **(J)** Masson staining illustrating the spatial distribution of newly formed bone within the scaffold. [Figure adapted from Wang et al. (2022)].

As key design variables, pore size and porosity regulate mass transport and mechanical cues, thereby profoundly shaping cellular behaviour in porous scaffolds (Silva et al., 2014). As Table 3 indicates, digital precision manufacturing via 3D printing can produce highly biomimetic trabecular-like porous scaffolds (Keaveny et al., 2001). Such biomimicry surpasses the conventional osteocyte spacing (24.1 ± 2.8 μm); pore diameters are generally larger, furnishing 3D room for cell proliferation, ECM deposition and vascularisation (Sugawara et al., 2005). Optimisation studies suggest that pore diameters of 400–600 μm are advantageous for osteogenesis and integration. Karageorgiou and Kaplan (2005) observed that pores ≤200 μm increased BMSC survival yet limited mass transport, restricting bone formation to the scaffold surface. Bonfield (2005) showed that 500 μm pores generating a 0.3–1.5 Pa shear gradient boosted HUVEC proliferation by 30%, confirming mechano-porous coupling. Arahira and Todo (2016) found that 400–600 μm pores in β-TCP/collagen scaffolds mediated tensile stress and specifically activated osteogenic pathways in BMSCs, supplying molecular evidence for mechano-regulated fate decisions. Addressing pore size-porosity-cell interplay, Wang et al. (2022) used SLM to fabricate trabecular-mimetic Ti-6Al-4V scaffolds to enhance osseointegration. Scaffolds with average pores of 300/400/500 µm in trabecular-inspired (ITS) and regular (RS) designs were fabricated and tested *in vitro* and *in vivo*. ITS scaffolds outperformed RS in cell proliferation, osteogenic differentiation and bone integration, with the 400 µm ITS exhibiting the highest potential (Figure 3). The work provides scientific guidance for designing bio-integrative titanium scaffolds and establishes a “structure–function” map for next-generation smart implants.

Porosity—the fractional void volume—within an optimal window enhances oxygen and nutrient convection through the scaffold (Takahashi and Tabata, 2004). Porosity modulates bone regeneration by: (1) creating continuous channels for gas/nutrient diffusion; (2) offering space for cell migration and ECM deposition; (3) guiding the topology of neovessel growth (Donnaloja et al., 2020). An appropriate porosity fraction is vital for defect repair; Wang Z. et al. (2017) demonstrated that matching scaffold porosity to trabecular bone (70%–90%) heightens osteoblast activity and depth of ingrowth, with wall curvature orienting cell polarity, connectivity tuning 0.8–1.2 Pa shear to upregulate osteogenic genes, and porosity >80% enhancing vessel density by 65% via VEGF gradients. An optimal porosity window thus improves angiogenesis and osteoconduction (Peng et al., 2019). (Ma et al., 2000) 3D-printed PET scaffolds and thermally modulated their pore size/porosity; trophoblast ED27 cells seeded directly on low-porosity, small-pore scaffolds formed fewer aggregates and showed reduced differentiation. Takahashi and Tabata 2004) found a positive correlation between porosity and BMSC osteogenesis in PET scaffolds, with 96.7% porosity yielding peak ALP activity and highest proliferation. Wei et al. (2016) showed that RVC-coated porous Ta enhanced adhesion, aggregation and proliferation both *in vitro* and *in vivo*; in canine models, 70%–85% porosity maintained >120 MPa strength while markedly improving defect repair. Nevertheless, excessive porosity diminishes scaffold mechanical strength, weakening structural support (Hudák et al., 2021). Hence, porosity must be judiciously tuned in 3D-printed scaffolds to balance mechanical competence with optimal bone-ingrowth capability.

Recent studies reveal that mechanical cues in the biomechanical micro-environment (0.5–2 Pa shear, cyclic compression) synergistically drive angiogenesis and osteogenesis via multi-scale mechanisms. Molecularly, shear stress activates the integrin-FAK axis, triggering cytoskeletal remodelling and AnxA6-mediated autophagic flux to enhance osteogenic differentiation; MC3T3-E1 cells subjected to 10 dyn cm^-2^ shear show elevated ALP and Col I, whereas AnxA6 knockdown suppresses autophagy and osteogenesis (Pei et al., 2022). Low shear (3–7 dyn cm^-2^) elevates Dll4 mRNA, fostering osteoblast–endothelial paracrine crosstalk; concomitant PI3K/Akt-MAPK activation markedly increases VEGF and Ang-1, expediting vessel maturation (Zhao Y. et al., 2022).

Bioreactor technologies likewise enhance coupled osteogenic-angiogenic outcomes. A bioreactor is generally defined as a device that modulates biological processes via mechanical means (Plunkett and O'Brien, 2011). As dynamic culture platforms, bioreactors are categorised *ex vivo* and *in vivo*; *ex vivo* units precisely impose 1–20 Pa shear on BMSCs to induce osteogenesis. Li et al. (2024) applied cyclic shear in an *ex vivo* bioreactor to composite hydrogel scaffolds, activating PI3K/Akt and MAPK pathways, up-regulating VEGF and Ang-1, and expediting vascular maturation. The *in vivo* bioreactor concept entails implanting the scaffold at a distant, non-defect site. Over time, mineralised tissue forms within the scaffold. This *in-situ* design fosters concurrent capillary and bone tissue formation. As most implants are cell-free, osteoprogenitors and endothelial cells proliferate in tandem, yielding vascularised bone. Initially relying on extravascular fluid for exchange, vessels eventually lumenise and mature, permitting blood perfusion that drives continued osteogenesis (Logsdon et al., 2014).

### 4.3 Bioactivity

The material used for the scaffold must possess sufficient bioactivity. The bioactivity of a material can be divided into biocompatibility, osteoconductivity, osteoinductivity, degradability, and angiogenic potential. Generally, 3D-printed artificial bone scaffold materials must facilitate osteoconduction, osteoinduction, and osteointegration. Their interconnected three-dimensional pore structure should meet the demands of nutrient and metabolic waste exchange while also guiding cell growth (Barba et al., 2018). The ability to foster cell adhesion and proliferation on the scaffold surface and within its pores is termed osteoconductivity (Guerrero et al., 2023). Osteoinduction denotes the capacity to induce osteogenesis by means of biological signaling, stimulating multipotent precursor cells to differentiate into osteoblasts (Xu et al., 2023). When an artificial bone scaffold and the bone form direct mechanical contact with no gradational relative motion and no fibrous tissue growth, the scaffold is considered osteointegrated (Guglielmotti et al., 2000).

Biocompatibility can be defined as the biological characteristic whereby a material remains relatively stable under the dynamic processes of the organism, tolerating the host systems without being rejected or destroyed (Overmann et al., 2020). The biocompatibility of scaffold materials encompasses supporting cell survival and function, while preventing cell apoptosis or immune responses (Tang et al., 2021). In 1968, Charnley and co-workers (Charnley, 1963) introduced a PTFE prosthesis placed between the femur and tibia for knee arthroplasty. In 2014, Williams (2014) confirmed the biocompatibility of PTFE, and, following exhaustive biosafety testing, it entered clinical use. Degradability is a critical design consideration for bone-graft scaffolds (Williams, 2008).

Biodegradability denotes the capability of a biomaterial to be progressively broken down by enzymatic (active) or hydrolytic (passive) processes *in vivo* or *in vitro* (Göpferich, 1996). An ideal scaffold should satisfy: (ii) a controllable degradation rate that synchronises with bone healing, thus preventing premature loss of mechanical support or prolonged foreign-body presence; (ii)biosafety, whereby degradation products are biocompatible and non-cytotoxic, avoiding local inflammation or systemic toxicity (Zhu et al., 2025). For critical bone defects, non-degradable implants may be left *in situ* to ensure lasting support; conversely, stabilised fractures treated with non-degradable scaffolds require secondary removal surgeries, heightening trauma, infection risk and recovery time (Mondschein et al., 2017). Consequently, smart scaffolds with tailored degradation kinetics are crucial to achieve the clinical goal of “single implantation, lifelong support.”

Composite scaffolds combine the biocompatibility advantages of disparate materials and optimise interfacial compatibility and synergistic reinforcement, thus advancing osteogenic-angiogenic performance and emerging as a research hotspot. For polymer/ceramic composites, surface coatings or alloying can improve the bioactivity of metallic bases, while chemical bonding or physical blending secures inter-material cohesion, thereby optimising interfacial compatibility. Yilmaz et al. (2025) incorporated fig-leaf extract into chitosan/HAp scaffolds; freeze-drying preserved high porosity (81.8%–85.9%), enhanced interfacial compatibility and elevated antioxidant and antibacterial efficacy (>90% inhibition). Synergistic reinforcement is key: polymers impart processability and toughness, whereas ceramics contribute bioactivity and osteoconductivity. In PLGA/HA scaffolds, HA osteoconduction promotes osteoblast adhesion and calcified nodule formation, whereas PLGA degradation dynamically adapts scaffold architecture to bone regeneration (Pereira Rodrigues et al., 2024). Fielding et al. (2012) 3D-printed β-TCP scaffolds combined with SiO and ZnO to create a composite construct. Implantation into rat femoral defects produced abundant type-I collagen and osteocalcin on the scaffold at 4 weeks. Compared with pure β-TCP, incorporation of SiO and ZnO markedly enhanced neovascularisation and new-bone formation. In metal/bioactive-coating hybrids, the metallic core supplies strength, whereas the coating confers immunomodulatory, osteogenic and angiogenic functions (Esen et al., 2016). Magnesium, an essential element in skeletal development, exhibits strong osteogenic and angiogenic potential among metals. Ma et al. (2020) functionalised 3D-printed porous Ta with Mg ions via polydopamine, creating an Mg-PDA-Ta scaffold that increased vascular density by 40% and accelerated osteocalcin deposition in rat femoral defects. Lai et al. (2019) 3D-printed a porous PLGA/TCP/Mg (PTM) scaffold from Mg powder, PLGA and β-TCP. In rabbit ulna defects, PTM scaffolds increased perfusion and angiogenesis within 4 weeks, showed well-formed vasculature at 8 weeks, and markedly enhanced new-bone formation and mechanics by 12 weeks. Gao et al. (2020) engineered a Mg-coated Ti-6Al-4V scaffold with augmented osteogenic and angiogenic potential. *In vitro*, the Mg coating markedly enhanced MC3T3-E1 proliferation, adhesion, ECM mineralisation and ALP activity, while up-regulating osteogenic genes. Fluorescence, micro-CT and histology confirmed significantly increased new-bone formation in rabbits *in vivo*. Moreover, the Mg-coated scaffold boosted HUVEC proliferation, adhesion, tubulogenesis and migration, upregulated HIF-1α and VEGF, and markedly improved angiogenesis.

Emerging 3D-printed technologies have propelled the field of bone tissue engineering, enabling the fabrication of patient-specific bone repair scaffolds from a variety of biomaterials. A key challenge in designing scaffolds for regenerative approaches is to ensure adequate vascularization, optimize their microstructure, mechanical characteristics, and material composition, and endow them with superior osteoconductivity and osteointegration. To achieve these goals, one can employ multiple materials and manufacturing technologies and also utilize seed cells, growth factors, and drug loading on artificial bone scaffolds as bioactive additives to enhance bone intraconstruct in the scaffold.

## 5 Loaded bioactive agents

Cells, growth factors, and scaffolds are often regarded as the three major components of tissue engineering (Fu et al., 2022). In the scaffold-induced bone repair process, the scaffold provides a template for bone tissue regeneration. By seeding cells, loading growth factors, and incorporating drugs onto a 3D-printed scaffold, it can be endowed with more comprehensive bionic functions. These scaffolds, containing cells, growth factors, and drugs, can be cultured *in vitro* to facilitate tissue formation and subsequently implanted into the damaged area, or they can be directly implanted into the damaged site *in vivo* to induce tissue or organ regeneration (Sparks et al., 2023). Introducing bioactive additives is an effective way to impart desirable biological properties to the inert scaffold surface. It can address the lack of bioactive substances on the scaffold surface, thereby enhancing vascularization and promoting the overall bone repair process. Researchers have consistently attempted to incorporate bioactive additives into scaffolds via physical doping and chemical modification to strengthen the vascularization capacity of 3D-printed scaffolds (Wang et al., 2025b; Min et al., 2024) To date, reported functionalization strategies for artificial bone scaffolds involve loading cellular components, active drugs, and growth factors into the scaffold, providing substantial support for osteogenesis and angiogenesis in tissue engineering. They can also incorporate copper ions and antibiotics to confer antimicrobial properties (Foroutan et al., 2019).

### 5.1 Cells

Bone is a highly regenerative tissue, characterized by a finely tuned balance between ECM formation mediated by osteoblasts and ECM resorption driven by osteoclasts, which facilitates ongoing bone remodeling (Robling and Bonewald, 2020). In recent years, strategies involving 3D-printed scaffolds loaded with cellular components for bone defect repair and vascular promotion have become a key research focus in tissue engineering. Specifically, researchers load artificial bone scaffolds with various autologous cell types—such as bone marrow mesenchymal stem cells (BMSCs), human mesenchymal stem cells (hMSCs), embryonic stem cells (ESCs), induced pluripotent stem cells (iPSCs), adipose-derived stem cells (ADSCs), endothelial cells (ECs), and human umbilical vein endothelial cells (HUVECs)—using different methods. The loaded progenitor cells adhere, grow, and differentiate, ultimately fostering bone and vascular formation.

Pittenger et al. (1999) were the first to demonstrate that BMSCs possess multipotent differentiation capabilities, giving rise to osteoblasts, chondrocytes, adipocytes, and more. Moreover, BMSCs exhibit immunoregulatory and immunosuppressive functions, reducing the likelihood of eliciting a host immune response (Duan et al., 2017). Regarding cellular sources, Scheinpflug et al. (2018) noted that BMSCs can be isolated from multiple tissues, with bone marrow, adipose tissue, and umbilical cord serving as favorable sources for regenerative medicine. In the study by Sparks et al. (2023), BMSCs were directly seeded onto a medical-grade ε-polycaprolactone-β-tricalcium phosphate (mPCL-TCP) scaffold. A pedicled cortical periosteal flap was then employed via an endogenous implantation method to treat large-segment tibial defects in sheep. Experimental findings revealed that the scaffold containing BMSCs demonstrated superior osteogenic and angiogenic outcomes. Beyond the robust osteogenic and angiogenic functions of BMSC-loaded scaffolds, researchers have also actively explored the incorporation of other cell types into scaffolds. Tsigkou et al. (2010) directly seeded hMSCs, HUVECs, and ESCs onto a 3D-printed porous scaffold. Compared with the control group, these scaffolds formed stable, long-lasting microvasculature *in vivo* in mice and successfully anastomosed with the host vascular system. McNamara et al. (2014) investigated a silk–hydroxycellulose composite scaffold with favorable mechanical properties, successfully seeding it with hMSCs. Their findings showed that the cell-laden composite scaffolds exhibited strong angiogenic capacity. Anada et al. (2019) fabricated a 3D-printed biomimetic bone composite structure based on octacalcium phosphate (OCP), HUVECs, and gelatin methacrylate (GelMA) hydrogels. *In vitro* results showed that OCP enhances the differentiation of HUVECs into osteoblast-like cells, whereas the HUVECs also facilitate the formation of new vasculature within the 3D-printed GelMA hydrogel (Nichol et al., 2010). In the study by Kuss et al. (2018), 3D-printed polycaprolactone–hydroxyapatite scaffolds were combined with human adipose-derived mesenchymal stem cells (ADMSCs) and HUVECs via hydrogel adhesion, forming a co-culture composite scaffold. Implantation into nude mice demonstrated that this composite scaffold facilitated anastomosis with the murine vasculature, and histological staining further confirmed that the co-culture of cells on the artificial bone scaffold supported both bone and vascular formation. This approach shows great potential for application in bone tissue engineering.

### 5.2 Growth factors

The formation of new bone and blood vessels is a complex, multistep process that is regulated directly or indirectly by numerous biological factors, such as angiogenic factors, osteogenic factors, cytokines, and growth factors (Alarcin et al., 2025).

Commonly employed growth factors in bone-tissue engineering include vascular endothelial growth factor (VEGF), bone morphogenetic protein-2 (BMP-2), and deferoxamine (DFO) (Wang et al., 2021). Loading various growth factors onto artificial bone scaffolds by different delivery strategies (Table 4) alters their release kinetics and effective concentrations, thereby influencing the scaffold’s capacity to induce bone and vessel formation. Because these growth factors are inherently unstable, their activity window after release is brief, whereas excessive expression or accumulation may provoke unwanted side effects (Silva and Lobo, 2020).

**TABLE 4:** Growth factors/bioactive drugs loading methods and release profiles.

Loaded Agent	Loading method	Carrier/Scaffold	Release characteristics	Time window of action	Dose optimization	References
BMP-2	Low-temperature 3D printing + layer-by-layer assembly	HA porous scaffold	Not specified	Mid-to-late osteogenic phase	Effective promotion of osteogenesis and angiogenesis	Chen et al. (2020a)
	Type I collagen hydrogel loading	PCL scaffold	Sustained release over 7–14 days	Early osteogenic differentiation (1–2 weeks)	Significant upregulation of ALP/Runx2 expression (7–14 days)	Park et al. (2015)
	Titanium nanotube (TiNT) encapsulation	3D-printed Ti scaffold	Sustained release (synchronized with bone remodeling)	Long-term osseointegration (>8 weeks)	Activates BMP/Smad pathway, enhances new bone formation	Ali et al. (2023)
VEGF	Gelatin microparticles (GMPs)	Matrigel scaffold	Sustained release over 3 weeks	Early vascularization phase (1–3 weeks)	Prolonged release significantly enhances vascularization efficiency	Poldervaart et al. (2014)
	PLGA microsphere encapsulation	Gelatin/Alginate/β-TCP scaffold	Sustained release within 10 days	Early tissue regeneration phase (≤10 days)	2-fold increase in HUVEC proliferation rate	Fahimipour et al. (2017)
DFO	Nanofiber layer thickening-controlled release	Silk fibroin (SF)-HA scaffold	63% cumulative release over 28 days	Dynamic vascularization (4–12 weeks)	Optimal osteogenic marker expression at moderate release rate	Fan et al. (2022)
DMOG	3D printing co-blending loading	MPHS scaffold (mesoporous bioactive glass/PHBHHx)	Sustained release over 4 weeks (zero-order kinetics)	Full-cycle repair phase (1–4 weeks)	Inhibits prolyl hydroxylase to stabilize HIF-1α, activating VEGF/BMP-2 synergistic pathway	Min et al. (2015)
Simvastatin	Thermosensitive PLGA-PEG-PLGA hydrogel	Porous Ti alloy scaffold (Sim-3DTi)	Sustained release (anti-tumor + osteogenic)	Integrated repair (anti-tumor and osteogenesis)	59%–77% tumor volume reduction, 40% bone density improvement	Jing et al. (2024)
Vancomycin	APTES grafting + electrostatic assembly	Porous Ta scaffold	Rapid release (early bacterial inhibition)	Early antibacterial phase (1–2 weeks)	Inhibits biofilm formation, promotes mineralized matrix production	Liu et al. (2022c)

Numerous studies demonstrate that BMP and VEGF play pivotal roles in osteogenesis and angiogenesis (Wang et al., 2010; Cha et al., 2021; Kawai et al., 2021; Zhou et al., 2015; Xu et al., 2022; Chang et al., 2014; Zhang et al., 2015). VEGF is regarded as the key signalling molecule for angiogenesis; it is upregulated in chondrocytes and osteoblasts within the fracture callus, and this elevation subsequently drives neovascularisation and bone regeneration (Freeman et al., 2021). Li Y. et al. (2021) fabricated 3D-printed porous Ti alloy scaffolds via electron-beam melting and loaded HUVECs together with VEGF onto the constructs using a hydrogel carrier. *In vivo* and *in vitro* studies in New Zealand white rabbits, complemented by histological and imaging analyses, confirmed that the composite scaffold enhanced angiogenesis and promoted bone repair. Poldervaart et al. (2014) used gelatin microparticles (GMPs) to modulate VEGF release and prolong its bioactivity; *in vitro* tests showed sustained VEGF delivery over 3 weeks. Endothelial-progenitor-cell migration assays confirmed the bioactivity of released VEGF. In a parallel *in vivo* study, Matrigel plugs containing EPCs and either rapidly released or GMP-sustained VEGF were implanted subcutaneously in nude mice; the sustained-release group exhibited significantly greater neovascularisation, indicating that extended VEGF delivery enhances vascularisation efficiency. Fahimipour et al. (2017) integrated VEGF-loaded PLGA microspheres into a gelatin/alginate/β-TCP scaffold; PLGA microencapsulation prevented burst release and provided sustained VEGF delivery during the first 10 days of regeneration, thereby meeting angiogenic demands. HUVEC proliferation on the scaffold doubled, confirming that VEGF fosters vascular-network formation and helps overcome the nutrient-supply bottleneck in bone regeneration.

BMP-2 is pivotal during embryonic skeletogenesis and in bone remodelling and integration in adulthood. Chen CY. et al. (2020) produced porous HA scaffolds via low-temperature 3D printing and layer-by-layer assembly, then loaded BMP-2 onto the scaffolds by simple adsorption. Results indicated that the composite scaffold exhibited excellent osteogenic and angiogenic capacity, effectively promoting new bone and vessel formation. However, the therapeutic efficacy of a growth factor is directly influenced by its loading dose. Sampath and Reddi (2020) reported that 1.4 µg BMP-2 was insufficient to induce bone union in rat femoral defects, whereas 11 µg achieved complete osseointegration. *In vivo*, the expression profiles of various BMPs orchestrate bone formation and skeletal growth (Halloran et al., 2020). Park et al. (2015) loaded BMP-2-bearing type-I collagen hydrogel onto strip-shaped PCL scaffolds; *in vitro*, ALP and Runx2 levels were two- and four-fold higher at day 7, and seven- and fourteen-fold higher at day 14, respectively, compared with scaffolds lacking BMP-2, confirming robust osteogenic promotion. Ali et al. (2023) tuned nanotube diameters (21–130 nm) on 3D-printed Ti surfaces via anodisation, encapsulated rhBMP-2, stabilised it with cryoprotectant, and demonstrated *in vivo* that released rhBMP-2 activated BMP/Smad signalling and markedly increased peri-implant bone formation.

Deferoxamine (DFO) is another potent vasculogenic agent (Potier et al., 2008). Mounting evidence shows that DFO elevates HIF-1α secretion, subsequently up-regulating VEGF and other angiogenic mediators, thereby enhancing vascular regeneration (Du et al., 2021). Fan et al. (2022) engineered an SF/HA scaffold loaded with DFO; thickening of the nanofibre layer markedly slowed DFO release, reducing cumulative 28-day release from 84% to 63% and enabling dynamic control of angiogenesis. Expression of osteogenic markers differed significantly among groups with varying DFO release rates, confirming that release kinetics directly influence osteogenic differentiation. In a rat bone-defect model, the scaffold with medium release achieved complete union within 12 weeks, raising bone density by 40% and doubling vessel density. However, DFO’s *in vivo* half-life is only 5–6 h, necessitating continuous supplementation to maintain therapeutic levels (Drager et al., 2017).

The use of a single growth factor often suffers from limited efficacy and inadequate spatiotemporal control. Synergistic multi-factor strategies—such as VEGF combined with BMP-2—have shown marked advantages. VEGF chiefly stimulates early angiogenesis, whereas BMP-2 drives osteogenic differentiation; MSCs not only differentiate into osteoblasts but also paracrinally release VEGF and BMP-2, establishing a positive-feedback loop (Liu Z. et al., 2022). Chen S. et al. (2020) used layer-by-layer assembly to co-load BMP-2 and VEGF onto cryogenic-printed HA scaffolds, enabling dual-factor controlled release and raising new-bone volume fraction by 60% vs. blank controls. Wang et al. (2018) found that VEGF and BMP-2 synergistically activated the p38-MAPK pathway and promoted osterix nuclear translocation, thereby enhancing osteogenesis and angiogenesis. Park et al. (2024) developed a CRISPR/Cas9-engineered tonsil MSC plus vitamin-D-PLGA scaffold system; BMP-2/VEGF overexpression combined with Mg(OH)_2_ slow release modulated M2 macrophage polarisation and markedly accelerated bone-defect repair. Thus, combinatorial strategies that integrate multiple factors, cell–material interactions and dynamic regulation substantially outperform single-factor applications.

### 5.3 Bioactive drugs

**FIGURE 4:**
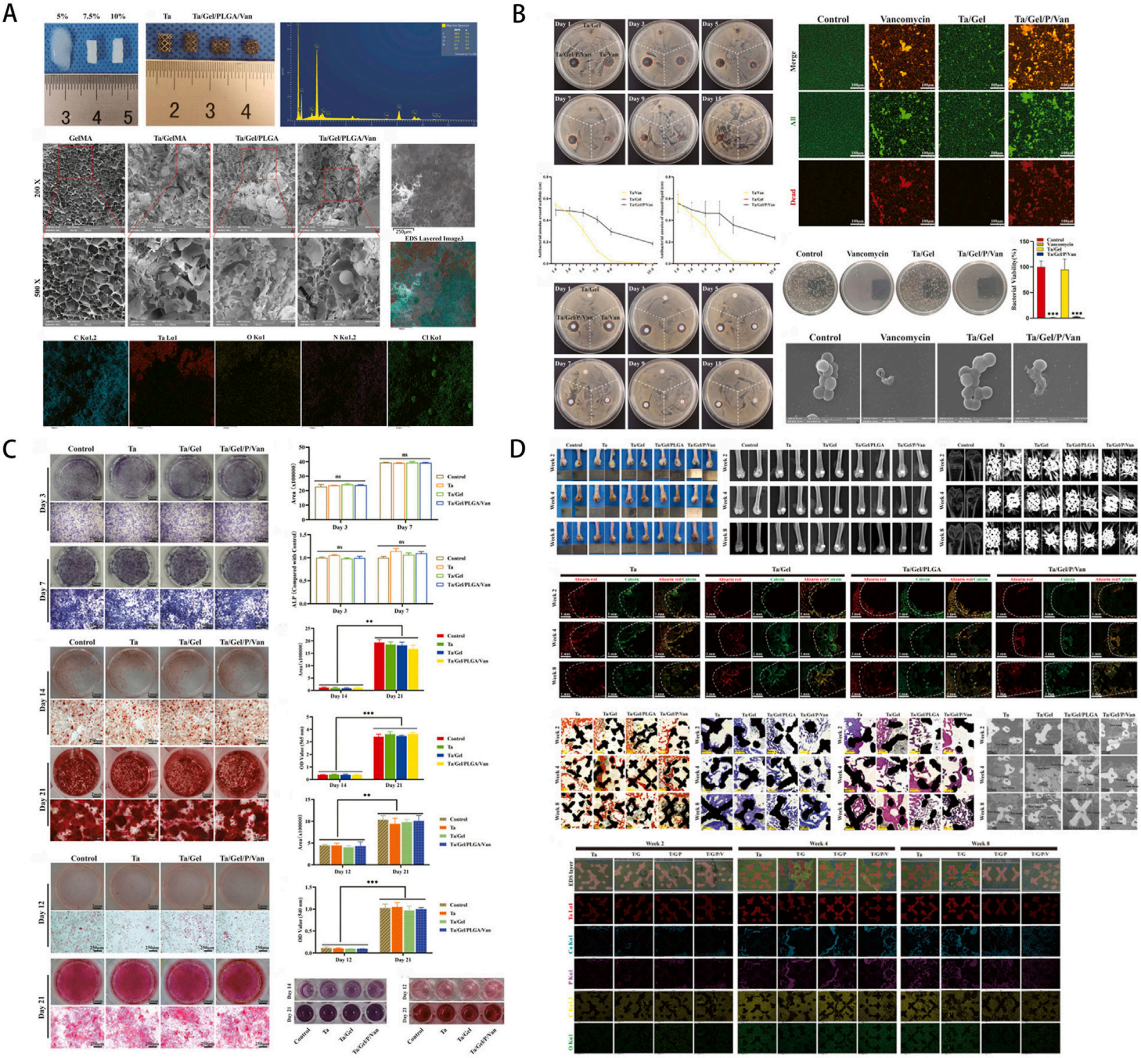
**(A)** Macroscopic morphology of hydrogels at varying concentrations and the Ta/Gel/PLGA/Van composite scaffold, with elemental composition and microstructural characterization via energy-dispersive spectroscopy (EDS) and scanning electron microscopy (SEM). **(B)** Antibacterial activity assessment through inhibition zone assays and live/dead bacterial staining, with SEM imaging revealing bacterial morphology alterations. **(C)** Osteogenic potential evaluation via histological staining, semi-quantitative analysis, and optical density (OD) measurements. **(D)** Bone conduction performance at 2, 4, and 8 weeks post-implantation, validated through macroscopic observation, X-ray imaging, micro-computed tomography (Micro-CT) analysis, fluorescence labeling, histological staining, and SEM examination. [Figure adapted from Qian et al. (2023)].

Small-molecule drugs are widely used in bone tissue engineering to enhance bone and vascular formation. Whether delivered via carriers or directly conjugated onto the scaffold, these drugs can endow the scaffold with more comprehensive functionality. Min et al. (2015) designed a 3D-printed composite scaffold (MPHS) composed of dimethyloxalylglycine (DMOG), mesoporous bioactive glass, and poly (3-hydroxybutyrate-co-3-hydroxyhexanoate). *In vivo* experiments showed that DMOG was released continuously from the MPHS scaffold over 4 weeks, significantly enhancing angiogenesis and osteogenesis at the defect site. Liu et al. (2016) used a rabbit tibial critical-size defect to assess a simvastatin/hydrogel-loaded 3D-printed porous titanium (pTi) scaffold. Compared with drug-free controls, the drug-loaded scaffold increased BV/TV 1.8-fold at 4 and 8 weeks and tripled neovessel density, confirming that simvastatin couples osteogenesis and angiogenesis via VEGF upregulation. Jing et al. (2024) employed a thermosensitive PLGA-PEG-PLGA hydrogel for controlled simvastatin release, inducing ferroptosis in osteosarcoma cells—TfR1 and NOX2 rose three- and four-fold, and xenograft tumours shrank by 59%–77%. In a rabbit condylar defect, the biomimetic porous scaffold upregulated BMP-2 several-fold, achieving tri-modal anti-tumour, osteogenic and angiogenic repair. Notably, its low-elastic-modulus design and bone–scaffold interlocking features indirectly enhance local angiogenesis by reducing stress-shielding effects, thereby further supporting the integrated “anti-tumor–osteogenic–vascularized” repair. Liu T. et al. (2022) fabricated a vancomycin-loaded porous tantalum scaffold; APTES grafting and electrostatic assembly with carboxymethyl-chitosan/vancomycin endowed antibacterial activity. The scaffold rapidly eradicated early-adhering bacteria, inhibited biofilm formation, promoted MSC mineralisation and osteogenic genes, while retaining tantalum’s structure and biocompatibility. In a rat infection model, the scaffold upregulated Runx2 and OCN and shifted macrophages toward an M2 phenotype, creating a pro-regenerative immune milieu. Qian et al. (2023) developed an integrated Ta/GelMA/PLGA/Van scaffold by encapsulating vancomycin in PLGA microspheres, embedding them in GelMA, and infilling a 3D-printed porous Ta lattice. *In vivo*, the scaffold released vancomycin continuously for 2 weeks and exhibited excellent biocompatibility, antibacterial action and osteointegration capability. This strategy offers a new avenue for one-step repair of infected bone defects, reducing re-operation trauma and costs, and holds significant clinical promise (Figure 4).

## 6 Axial vascular prevascularization in artificial bone scaffolds

Insufficient vascularisation is the primary reason for treatment failure in large-segment bone defects and for the limited clinical use of complex biomaterials. During scaffold-guided tissue regeneration, establishing an early, functional blood supply is the critical rate-limiting step for successful regeneration and repair (Spater et al., 2020). Das and Botchwey (2011) showed that without active blood perfusion, metabolite diffusion within a tissue-engineered scaffold is limited to 100–200 μm, which directly compromises cell survival and function in the scaffold core. To address this, they proposed a regeneration-matched axial vascularisation strategy: pre-formed axial channels guide host vessels to grow directionally into the scaffold, while vascular progenitor cells are delivered to stabilise the early neovasculature. Pre-vascularisation of the scaffold is achieved by implanting a vascular pedicle into its interior. The implanted pedicle anastomoses with recipient vessels at the implantation site, establishing direct blood perfusion within the scaffold (Eweida et al., 2014). The concept of scaffold-guided vascularisation builds on our understanding of how native tissues acquire their blood supply and on the use of modern reconstructive surgical techniques to replicate that supply (Yang H. et al., 2022).

### 6.1 Perforator arteriovenous loop

An arteriovenous loop (AVL) is created by directly anastomosing an artery and a vein *ex vivo* or *in vivo* to form a closed ring, thereby establishing an initial zone of high perfusion inside the scaffold. The pressure gradient between high-pressure arteries and low-pressure veins drives blood flow through the loop, giving nascent vessels optimal mechanical cues and nutrient delivery. This concept was initially proposed by Erol and Sira (1980), who successfully demonstrated in a rat model that axially vascularized skin could be induced via an AVL, and it was later expanded by Tanaka et al. (2000). In animal models, a superficial artery and vein are typically anastomosed to form an AV loop, which is then perforated through a compartment containing bioactive materials to yield vascularized bone tissue during the incubation period (Weng et al., 2021). Hofer et al. (2003) investigated the role of AVL in scaffold pre-vascularisation and assessed how growth-chamber volume affects tissue formation. They enlarged the AVL chamber from 0.45 mL to 1.9 mL, routed the loop through a PLGA scaffold that supplies mechanical support and cell-adhesion sites, and implanted the construct into four groups of rats (n = 5 each) for 6 weeks. The AVL-PLGA system proved osteo- and angio-genic and scalable: tissue yield increased by 73% (0.57 g vs. 0.33 g) under identical loop conditions, while gradual PLGA degradation (77.5% at 8 weeks) created space for new tissue and enabled scaffold-to-tissue transformation, offering a controllable framework for large-defect repair. Other investigators have shown that a hypoxia gradient drives orderly neovascularisation in the AVL model. Yuan et al. (2017) analysed the spatial distribution of HIF-1α and its correlation with angiogenesis. Thirteen rats (groups of 4, 5, 4) received femoral AV anastomoses embedded in fibrin chambers; HIF-1α, macrophages (ED1) and endothelial cells (lectin) were examined on days 7, 10 and 14. Results showed close coupling between HIF-1α distribution and vascularisation: positivity increased from the central loop toward the periphery (days 7–10), driving outward vessel growth, whereas global HIF-1α declined at day 14 as mature vascular zones expanded, confirming hypoxia as a key angiogenic driver. Macrophages carried most HIF-1α (>50% positive cells), suggesting that steering their polarisation (e.g., toward M2) could improve angiogenic efficiency. Day 10 marked a surge in vessel growth (peripheral HIF-1α peak); administering pro-angiogenic factors such as VEGF at this time could maximise efficacy. Horch et al. (2014) were the first to merge tissue-engineering with the AVL model, microsurgically anastomosing the lingual artery and an internal-jugular branch to restore large radial and tibial defects after osteomyelitis debridement. Acting as an axial vessel, the AVL combined with cancellous bone, fibrin glue and β-TCP/HA scaffolds to enhance bone regeneration. When the scaffold was transplanted into a mandibular defect after four tumour resections, CT at 6 and 24 months revealed pronounced new bone around the graft. Biopsies at 8 and 24 months showed vascular-rich fibrous tissue with woven bone at 8 months, and abundant mature bone with patent graft vessels at 24 months; the defect fully healed and the patient experienced no donor-site complications. The study confirms the long-term feasibility and stability of AVL technology for reconstructing complex mandibular defects.

### 6.2 Perforator arteriovenous bundle

An arteriovenous bundle (AVB) is an alternative vascularisation strategy in which an artery and vein are tied together without direct anastomosis. This configuration relies on the reconstruction of local tissue microcirculation and promotes capillary network formation. In the AV bundle model, an unbranched AVB is threaded through a custom chamber filled with osteo-inductive material to obtain a vascularised bone graft. Unlike the AVL model, the AVB approach requires no vascular anastomosis. This reduction lowers the risk of thrombosis and aneurysm formation. Because no extra venous segment is transplanted, the method simplifies AVL surgery, although it yields less fibrous tissue (Wu et al., 2017). Successful applications of the bundle technique have already been reported. Houben et al. (2021) orthotopically transplanted allogeneic tibial segments containing an AVB into matched tibial defects in recipient pigs. In the test group the native nutrient vessels were anastomosed and an autologous AVB was added inside the medullary canal, whereas controls received only the native vascular repair. At 20 weeks post-op, IL-2 expression was markedly lower in the AVB group, with significantly less bone necrosis and fibrosis. These findings indicate that an autologous AVB enhances vascularisation, improves graft vitality and mitigates rejection-related damage. Li et al. (2018) inserted a femoral AVB into the central tunnel of a 3D-printed PLGA/β-TCP scaffold and incorporated sustained-release rhBMP-2 microspheres to build a pre-vascularised bone-regeneration composite. After implantation into rabbit thigh, micro-CT at 4 weeks showed higher mineral density in the SBV group; at 12 weeks bone-area fraction reached 70.76% in SBV versus 47.84% in SB. Angiography revealed denser peripheral and central vessels in SBV, overcoming central graft necrosis. Han et al. (2014) combined a printed β-TCP scaffold with BMSCs, periosteum and a great-saphenous AVB (experiment) versus scaffolds lacking periosteum/AVB (control). Four weeks after implantation into rabbit limbs, capillary density was 14 ± 1.48 per field in the AVB group versus 7.9 ± 1.57 in controls (P < 0.05). BV/TV, trabecular thickness and number (14.82%, 43.78 μm, 5.32/mm^2^) were all higher in the AVB group, with significantly more new bone in the scaffold. These results verify AVB pre-vascularisation as a feasible technique for repairing large-segment bone defects.

### 6.3 Perforator arteriovenous flow-through, venous bundle and muscle pouch

Deng et al. (2020) were the first to combine the Masquelet technique with an arteriovenous flow-through procedure (muscle-flap AV anastomosis) in an emergency setting (<6 h post-trauma). Seventeen patients with 3–13.6 cm (median 5.73 cm) Gustilo-Anderson IIIA/B/C defects received antibiotic cement optimised by adding 3 g vancomycin per 40 g cement. The muscle-flap artery and vein were anastomosed at the defect to form an AV flow-through, and the AV-cement unit was inserted into the gap. The robust blood supply enhanced membrane vascularisation, raising CD31^+^ microvessel density 3.2-fold. Infection fell from the reported 25%–50% to 0%, and amputation from 16%/42% (IIIB/IIIC) to 0%.Recently, investigators have pre-built AV conduits inside osteoconductive matrices. Arkudas et al. (2010) 3-D-printed cage-like Ti-6Al-4V scaffolds. HA/β-TCP particles plus fibrin glue were packed inside, and an AV flow-through was threaded through the cage. 3-D angiography at weeks 2 and 8 showed vessel area in the AV group far exceeding controls (256.3 ± 78.9 μm^2^ vs. 92.2 ± 40.1 μm^2^, p < 0.05). This indicated more mature vasculature. Combining cells, growth factors and AV loops can yield large, vascularised tissue-engineered bone flaps. Microsurgical implantation into bone defects achieved notable repair. The study further confirmed feasibility for large-defect repair (Arkudas et al., 2007).

**FIGURE 5:**
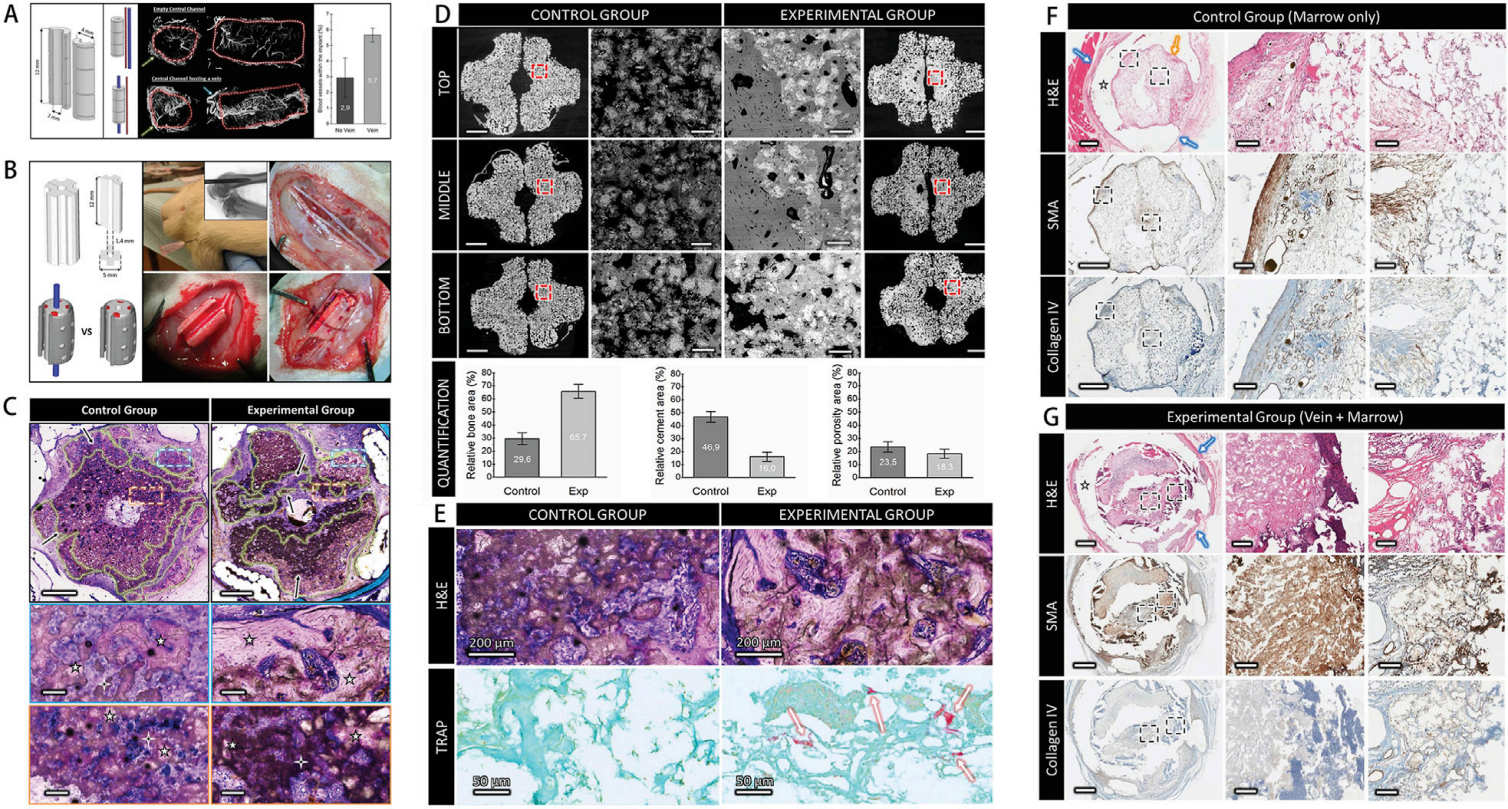
**(A)** CAD-modeled scaffold design with Micro-CT cross-sections showing vascular distribution. **(B)** Scaffold fabrication process, including intravascular positioning, bone marrow aspiration, and assembly. **(C)** SEM analysis of the scaffold’s top, middle, and bottom regions, revealing microstructural variations. **(D)** H&E staining showing increased bone formation in the experimental group compared to controls. **(E)** Alizarin Red S and Toluidine Blue staining highlighting mineralized tissue, with arrows indicating bio-ceramic degradation and circles marking lamellar bone. **(F,G)** Immunohistochemical analysis of ECM distribution and tissue remodeling, comparing the experimental (bone marrow + venous perfusion) and control (bone marrow only) groups, adapted from Charbonnier et al. (2019).

Although AVL, AVB and AV flow-through markedly enhance osteo-angiogenesis, microvascular anastomosis increases surgical complexity. Charbonnier et al. (2019) fabricated a 3D-printed, highly erosive bioceramic artificial bone scaffold using only a single vein combined with bone marrow aspirate. The scaffold was positioned around the intact femoral vein (with preserved blood flow) and subsequently implanted subcutaneously in five rats as the experimental group. In contrast, for the control group, the scaffold was placed adjacent to the blood vessel, relying exclusively on external vascular ingrowth. Eight weeks post-operation, micro-CT and immunohistochemical analyses were performed to evaluate the effects of central veins on bone formation, focusing on α-SMA (vascular maturity), type IV collagen (basement membrane), and TRAP (osteoclastic activity) (Figure 5). The results demonstrated that the osteogenic induction level in the control group ranged from 9% to 26.6%, whereas after induction with a single venous trunk, the osteogenic level increased significantly to 66% ± 6%. Notably, substantial new bone formation was observed in the venous perfusion group. Furthermore, the density of newly formed blood vessels doubled (5.7% ± 0.4% versus 2.9% ± 1.3%, p < 0.05), with the network penetrating the scaffold and accelerating the formation of vascularized bone tissue. This study represents the first evidence that a single vein can promote vascularization and bone tissue regeneration via an intrinsic vascularization axial perfusion mechanism.

## 7 Vascularized tissue flap transplantation

**FIGURE 6:**
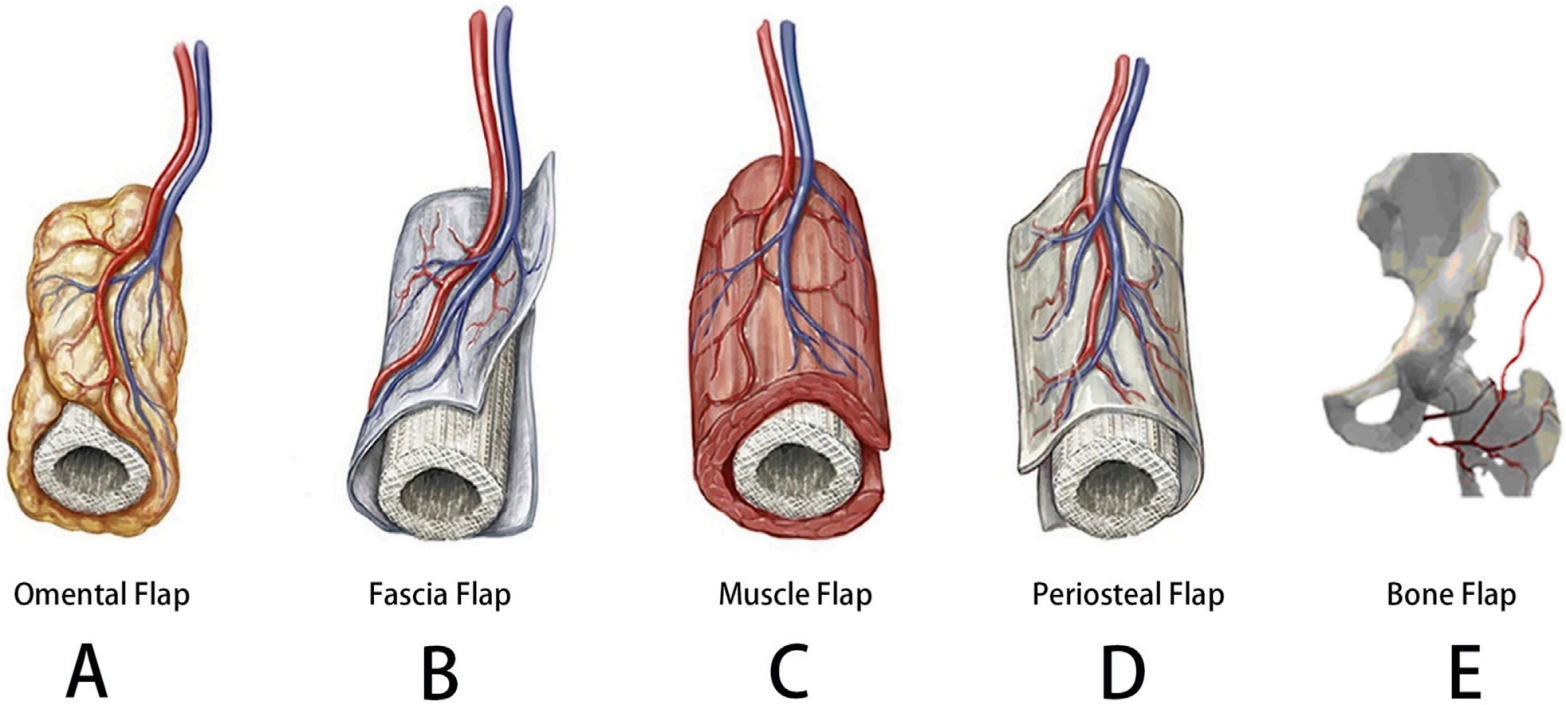
Vascularization of the scaffold facilitated by the combination of vascularized tissue flaps and artificial bone scaffolds. [Figure adapted from Sparks et al. (2019)]. **(A)** Omental **(B)** fascia flap **(C)** muscle flap **(D)** periosteal flap **(E)** bone flap.

Compared with pre-vascularisation by perforator axial-vessel transfer, pedicled flap transplantation saves *in vivo* pre-vascularisation time and, via microsurgical anastomosis, preserves the flap’s perfusion and viability more completely, thereby supplying abundant blood to support bone regeneration. Highly perfused tissue flaps—such as omental, fascial, muscular, periosteal and bone flaps—can serve as vascular beds to augment scaffold vascularisation (Beier et al., 2004). Vascular-dense flaps are wrapped peripherally around the bone scaffold and the construct is implanted into the defect site (Khouri et al., 1991). To facilitate bench-to-bedside translation and spare patients a second operation, pedicled omental, fascial, muscular or periosteal flaps can be transferred inside or around the scaffold, achieving *in-situ* vascularisation (Figure 6). (Sparks et al., 2019). Such pedicled flaps are transplanted to the defect by microsurgery and envelop the scaffold, thereby establishing its vascular supply. The rationale is that flaps already containing a vascular network, progenitor cells and growth factors effectively stimulate scaffold vascularisation and tissue regeneration. This strategy not only fosters neovascularisation and osteogenesis but also permits flap transfer onto the scaffold, thus enhancing bone healing at the defect. For regeneration of large-segment defects, diverse flaps are used to vascularise scaffolds that have been preloaded with cells and growth factors before implantation. These strategies aim to pre-engineer vascularised tissue *ex vivo*, improving post-implant anastomosis with host vessels and boosting cell viability and new-bone formation.

### 7.1 Fascial flap

**FIGURE 7:**
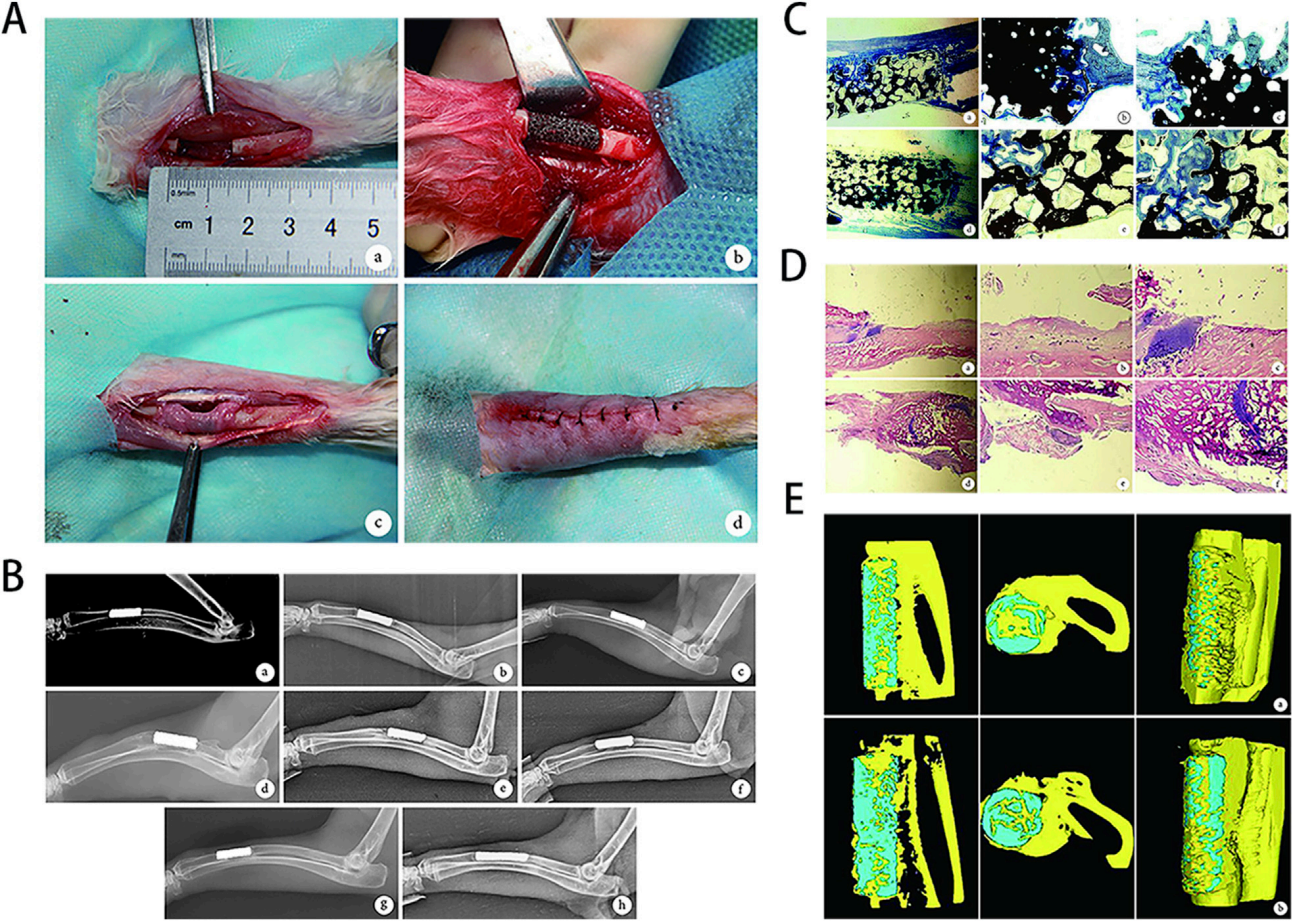
**(A)** Creation of the rabbit radius large segmental bone defect model and surgical procedure. **(B)** Postoperative X-ray images for both groups. **(C)** Postoperative histological analysis with Toluidine Blue staining and **(D)** HE staining. **(E)** Micro-CT images at 16 weeks postoperative, with tantalum metal shown in light blue and newly formed bone in yellow. [Figure adapted from Wang H. et al. (2017)].

Fascial flaps, given their dense vascular network and excellent permeability, are highly suited for enhancing vascularization in tissue-engineered bone. In experimental studies, fascial flaps in combination with artificial bone scaffolds have successfully induced bone and vascular regeneration, yielding notable results (Cherubino et al., 2016). Fan et al. (2014) created 20 mm bilateral tibial segmental defects in 45 rhesus monkeys and fabricated 3D-printed porous β-TCP scaffolds, seeding the scaffolds with MSCs and wrapping them with a vascular bundle plus pedicled fascial flap (experimental), while blank scaffolds served as controls, and performing quantitative vascular-perfusion analysis at 4, 8 and 12 weeks post-implantation, which revealed a vascular-density of 521.5 ± 77.8 units in the experimental group versus only 98.4 ± 20.3 in controls at 12 weeks, demonstrating markedly faster and denser angiogenesis—especially in the scaffold core—in the experimental group, while histology showed complete scaffold resorption, abundant mature trabeculae and marrow reconstruction at 12 weeks, whereas controls degraded slowly with new bone restricted to pore edges, indicating that pedicled fascial flaps markedly promote functional vascularised bone regeneration. Wang H. et al. (2017) fabricated 3D-printed porous tantalum scaffolds and created 1.5 cm segmental radial defects in 60 rabbits, wrapping the rods with 30 × 20 mm pedicled fascial flaps for the experimental group and leaving bare scaffolds as controls, and assessed X-ray, HE/toluidine-blue staining, three-point bending and micro-CT at 16 weeks, which demonstrated that fascial wrapping raised load-to-failure by 16.8% and bending strength by 16.2%, while micro-CT showed BV/TV at 32.63% ± 3.56% versus 25.07% ± 4.34% in controls, The new bone volume fraction was significantly higher in the experimental group than in the control group (30.2% increase), confirming its ability to promote bone ingrowth (Figure 7). Wrapping porous tantalum scaffolds with pedicled fascial flaps accelerates perfusion, boosts bone ingrowth and improves mechanics, enabling efficient defect repair, making it particularly suitable for poorly vascularised segmental defects and offering a clinically practical (easy flap harvest) yet effective solution.

### 7.2 Muscle flap

Owing to its abundant blood supply and mesenchymal stem cell source characteristics, muscle tissue plays a crucial role in fracture healing (Jiang et al., 2024). It is effective for the treatment of extensive bone defects caused by infection or trauma, as the muscle microenvironment can maintain bone formation via growth factor stimulation, a feature critical for the healing of large bone defects (Chen et al., 2004). In the vascularization of artificial bone scaffolds, muscle flaps are frequently employed, with the latissimus dorsi flap being a commonly used option to wrap the scaffold. From the perspective of bone reconstruction, muscle tissue offers a richer capillary network for bone defects than fascial tissue (Li et al., 2009). Warnke et al. (2004) implanted a growth-factor/bonemarrow-filled titanium cage into a patient’s latissimus dorsi; after 7 weeks of in-muscle vascularisation, the construct was transferred to a mandibular defect and achieved successful repair. Mehta et al. (2018) covered 39 Gustilo IIIB tibial fractures with either muscle or fascial flaps. Imaging showed markedly faster bone union in the muscle-flap group during the first 6 months. To date, pedicled-muscle-wrapped scaffolds have repeatedly proven effective for segmental bone loss. In a rat model, Khouri et al. (1991) placed an adductor muscle island flap with demineralised bone matrix (DBM) into a silicone chamber; within 10 days the osteogenin/DBM-treated flap transformed wholly into cancellous boneTerheyden et al. (1999) implanted xenogenic bone mineral plus BMP-7 into a latissimus muscle pouch in pigs and found that muscle-flap wrapping effectively induced scaffold osteogenesis and vascularisation. Pedicled-muscle wrapping offers notable advantages: muscle flaps are widely available, richly perfused and supply an axial vessel. Nevertheless, despite promising studies and clinical experience, widespread adoption faces obstacles. Chief concerns include implant failure, exposure and infection at the defect site. Despite these limitations, the muscle-flap–scaffold strategy has shown feasibility for bench-to-bedside translation in large-defect repair.

### 7.3 Omental flap

**FIGURE 8:**
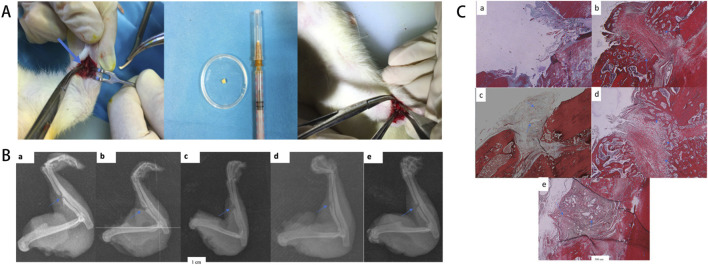
**(A)** Schematic representation of the bone defect model creation and transplantation of decellularized mesenchymal stem cell-loaded membrane scaffolds into the defect site. **(B)** Radiological images of the different groups, from left to right: control group, membrane group, membrane + mesenchymal stem cell group, membrane + PRP group, and membrane + mesenchymal stem cell + PRP group. Blue arrows indicate the defect regions. **(C)** Histological staining showing osteogenic potential of the materials in the *in vivo* experiment. [Figure adapted from Emet et al. (2021)].

The angiogenic properties of the omentum were recognized centuries ago, and due to its high flexibility and dense vascularity, it is widely employed for axial vascularization of artificial bone scaffolds (Meza-Perez and Randall, 2017). Compared with muscle tissue, the omentum is thinner, more elastic, highly vascularized, and contains precursor cells that support osteogenic differentiation (Naujokat et al., 2020). Naujokat et al. (2019) explored the concept of in-body cultivation by using the greater omentum as a bioreactor for bone-tissue engineering. In their study, scaffolds loaded with BMPs and bone-marrow aspirate were wrapped in omental or periosteal flaps and implanted in pigs. Histological and radiological analyses showed marked bone-density gains at 8 and 16 weeks, with the periosteal flap yielding superior osteogenesis. The work highlights the potential of bioreactor cultivation as an *in-situ* bone-regeneration strategy, exploiting endogenous regenerative capacity to boost scaffold vascularisation and osseointegration. In a rat study, Emet et al. (2021) combined decellularised omental flaps, platelet-rich plasma and MSCs with bone scaffolds and implanted them into bilateral radial defects to assess healing. After 6 weeks all animals showed viable trabeculae; imaging and histology indicated preserved cell vitality on the decellularised flap and significantly better defect healing than controls (Figure 8). Omental flaps, being highly pliable, are well-suited to microsurgical reconstruction. Birkenfeld et al. (2019) wrapped hydroxyapatite scaffolds with the greater omentum, loaded them with rhBMP-2, and implanted them into mandibular defects in rabbits. Histology and fluorescence microscopy confirmed new bone, neovessels and connective-tissue formation. As a highly vascular peritoneal tissue, the omentum offers outstanding infection resistance, making it particularly suitable for treating osteomyelitis associated with severe bone loss (Kamei et al., 2010). Moreover, the omentum’s contribution after transfer is tightly linked to its potent angiogenic potential, with resident MSCs playing a key part. Studies indicate that the omentum’s striking angiogenic traits may stem from its markedly higher expression and production of vascular endothelial growth factor compared with other tissues (Naujokat et al., 2020).

### 7.4 Periosteal flap

**FIGURE 9:**
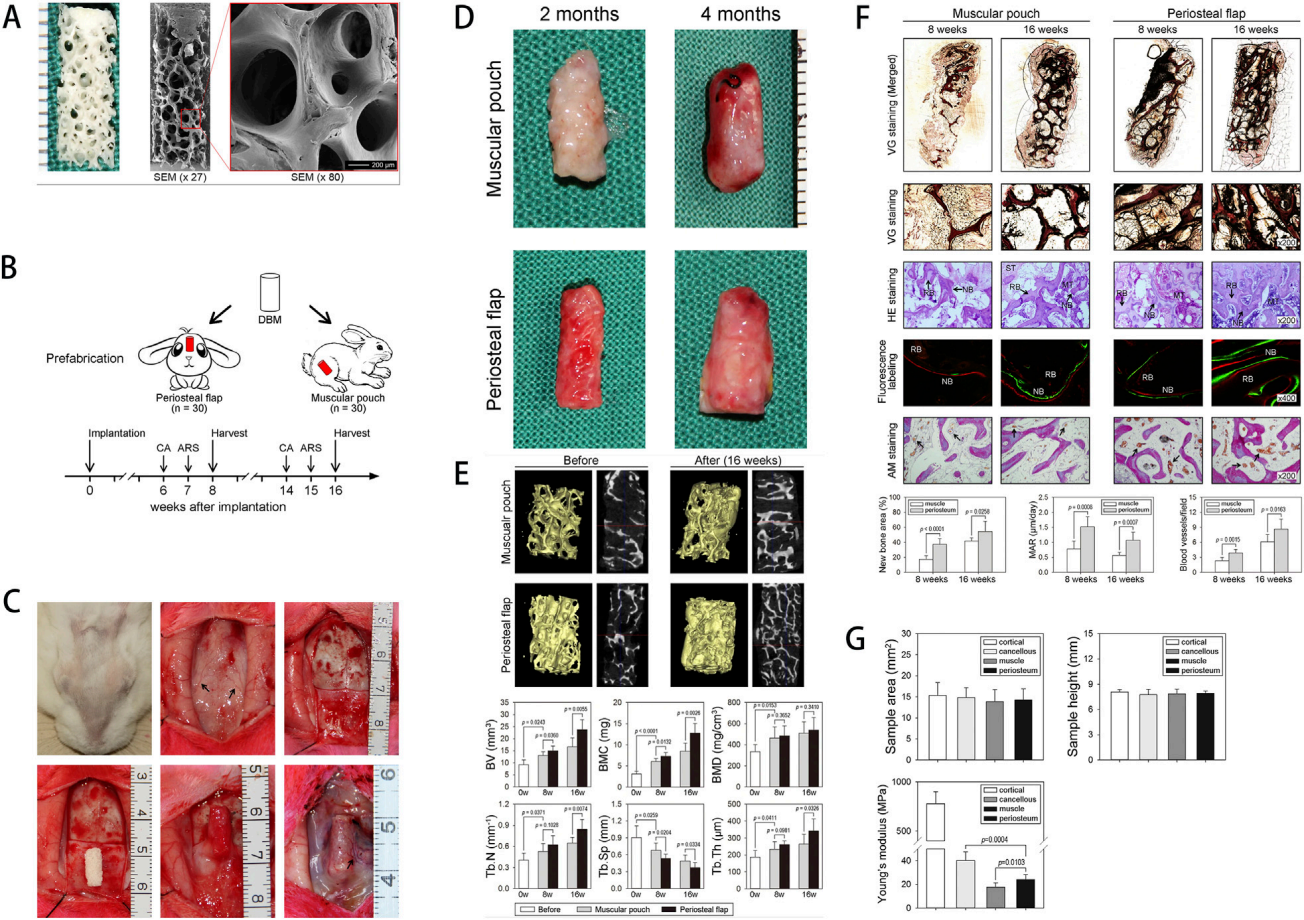
**(A)** Macroscopic and SEM images of the DBM scaffold. **(B)** Experimental groups and time points, comparing pre-vascularized muscle flaps with vascularized periosteal flaps combined with scaffolds. Newly mineralized bone was labeled one and 2 weeks before sampling. **(C)** Exposure of the supraorbital vessels (black arrow) on the rabbit calvarium, with a vascularized periosteal flap surrounding the DBM scaffold. **(D)** Comparison of muscle bag versus vascularized periosteal flap strategies, with the latter resulting in more complete bone formation. **(E)** Micro-CT reconstruction before and after DBM implantation, with quantitative analysis of BV, BMC, BMD, Tb.N, Tb. Sp, and Tb.Th. **(F)** Histological staining revealing newly formed bone and blood vessels. **(G)** Analysis of scaffold cross-sectional area, height, and Young’s modulus, indicating that the bone graft formed using the vascularized periosteal flap combined with the artificial bone scaffold exhibits mechanical properties comparable to cancellous bone. [Figure adapted from Huang et al. (2017)].

The periosteum is a thin, bilayered tissue with an abundant capillary network and nerve supply, composed of an outer fibrous layer and an inner cambium layer. It is capable of inducing bone repair and promoting growth of cancellous and cortical bone (Roberts et al., 2011; McLoughlin et al., 2025). It plays a pivotal role in fracture healing and in the repair of large-segment bone defects. During microsurgical procedures, obtaining an intact periosteal flap is critical for preserving the periosteum’s osteogenic potential, ensuring it retains its regenerative ability throughout transplantation. A pedicled periosteal flap contains MSCs, bone matrix and multiple growth factors, markedly enhancing new-bone formation; this superiority explains why periosteum outperforms muscle in osteogenesis (Finley et al., 1978). Huang et al. (2017) assigned 60 rabbits to a test group—DBM scaffold wrapped with a 1.5 × 1.5 cm pedicled periosteal flap based on supraorbital vessels—and a control group with the scaffold implanted in a thigh muscle pouch (Figure 9); Micro-CT, histology (HE, VG) and vessel-density assays were performed at 8 and 16 weeks. Bone volume in the periosteal group was 1.62-fold and 2.56-fold baseline at weeks 8 and 16, versus 1.40-fold and 1.79-fold in controls. HE staining revealed new-bone area 2.40 × and 1.45 × greater than controls at 8 and 16 weeks, respectively. These data confirm that a pedicled periosteal flap induces scaffold osteo-angiogenesis effectively and more efficiently than muscle-pouch wrapping. Strategies that vascularise bone scaffolds with periosteal flaps to trigger repair are now widely employed experimentally. Tatara et al. (2016) implanted autologous-bone/synthetic-ceramic scaffolds onto sheep rib periosteum, allowed vascularisation, then transferred them to mandibular defects for reconstruction. Histological and radiological assessments confirmed abundant bone formation *in vivo* and successful mandibular repair. Redenski et al. (2021) pre-vascularised decellularised bone matrix (DCB) scaffolds *in vitro* using a composite pedicled bone-and-soft-tissue flap co-cultured with endothelial and support cells before implanting them into rat tibial defects. The composite scaffold was subsequently transplanted to the tibial defect for repair. Results showed marked enhancement of angiogenesis and osteogenesis within the defect. The scaffold not only bridged the bone gap but became surrounded by regenerated muscle fibres, significantly improving biomechanical strength. In Xu et al. (2022) work, porous β-TCP scaffolds were pre-vascularised for 3 weeks in lateral-tibial muscle pouches, then transplanted with a pedicled periosteal flap into large tibial defects. Non-vascularised β-TCP scaffolds served as controls. Imaging and histology demonstrated that the pre-vascularised, periosteum-wrapped scaffolds markedly enhanced *in-situ* angiogenesis and osteogenesis relative to controls. The study confirms that combining muscle-pouch pre-vascularisation with periosteal pedicle transfer not only strengthens osteogenesis but also prevents ectopic ossification. This approach holds broad promise for future bone-defect repair investigations.

### 7.5 Bone flap

The complex three-dimensional anatomy of bone makes bone defect repair highly challenging. Not only can 3D-printed technology customize biomimetic scaffolds for precise bone defect repair, it can also integrate 3D imaging into preoperative or intraoperative navigation systems, enabling the accurate transplantation of vascularized bone flaps to defect sites (Gokdogan, 2022). In clinical practice, vascularized bone flaps, with their abundant blood supply, not only prevent potential ischemic necrosis following biomimetic scaffold implantation but also feature smaller surgical wounds and less bleeding. In treating femoral head necrosis, Cai et al. (2020) conducted a comparative study in 28 patients with avascular necrosis of the femoral head. One group did not use 3D-printed–assisted navigation with a vascularized iliac bone flap, while another group of 15 patients did Radiological and statistical analyses showed that combining 3D-printed navigation with a vascularized iliac bone flap in treating femoral head necrosis enabled more precise localization of the necrotic area, shortened operative time, reduced intraoperative bleeding, and improved early postoperative hip function. Throughout this process, the integration of 3D-printed technology with vascularized bone flap transplantation not only enhanced surgical precision but also expedited patient recovery. This technique shows promising potential for clinical bone defect repair in the future. Zhao et al. (2019) explored vascularized iliac bone flap transfer as a hip-preserving strategy for femoral head osteonecrosis, emphasizing its role in restoring blood supply, decompressing the femoral head, and providing structural support. Their study outlined a stepwise surgical approach, including necrotic bone debridement and implantation of both cancellous bone and a vascularized iliac bone flap. Compared to alternative treatments such as core decompression or nonvascularized bone grafting, this technique offers superior revascularization and mechanical stability, making it a viable option for patients in the early to mid-stages of osteonecrosis. These findings highlight the importance of vascularization strategies in bone tissue engineering, reinforcing the need for bioactive, vascularized scaffolds in the repair of large bone defects.

### 7.6 Osteoimmunomodulation strategies

Pedicled flaps markedly optimise scaffold performance by providing a physical barrier and immunomodulation: an intact vascular endothelium separates the scaffold from host immunity, minimising foreign-body reactions. Implantation typically provokes inflammation that recruits immune cells, especially monocytes. Recruited monocytes may differentiate into pro-inflammatory M1 or anti-inflammatory M2 macrophages. A biocompatible scaffold should bias monocyte polarisation toward the anti-inflammatory M2 lineage (Bian et al., 2023). Tailoring scaffold immunomodulation can therefore enhance osseointegration—facilitating intercellular signalling while remaining biocompatible and non-toxic to surrounding tissue (Cen et al., 2025). Moreover, MSCs within pedicled flaps secrete TSG-6 and IL-10, driving macrophage transition from M1 to M2, suppressing TNF-α and IL-1β, remodelling the immune milieu and synergistically boosting osteogenic and angiogenic outcomes. During angiogenesis, host endothelial cells activate VEGF/FGF pathways via HIF-1α to start branching; post-implant inflammation can accelerate this sequence. Tie2^+^ monocyte-macrophages (TEMs) then enhance endothelial proliferation and lumen maturation via the ANGPT2–Tie2 axis (Tai et al., 2025). Paracrine M2-derived extracellular vesicles deliver miR-126-5p, dampen fibroblast activation ((α-SMA decreased by 50%)) and upregulate VEGFA/IGF-1, thereby optimising the niche for functional vasculature (Zhang et al., 2025).

Nonetheless, implants risk immune rejection and fibrosis: excessive M1 polarisation (CD86^+^ > 80%) promotes soft-tissue overgrowth that outpaces osteogenesis, causing fibrous encapsulation and failure (Spiller et al., 2015). To overcome these hurdles, multiscale interventions should target: (1) controlled release of anti-fibrotic agents such as TGF-β inhibitors; (2) optimisation of ectopic culture duration to synchronise pedicle maturation with bone accrual; (3) mechano-immune co-stimulation—cyclic loading to trigger SMC–EC contact-dependent signals (eNOS/NF-κB) and shear-responsive vascular homeostasis (Hong et al., 2013). Future work should integrate personalised immune profiling, interface biomimicry and 4-D-printed smart scaffolds with degradable, mechano-responsive, spatiotemporal release to shift bone regeneration from structural repair to functional restoration.

## 8 Limitation

Despite the advancements in 3D-printed vascularized bone scaffolds, several challenges remain in terms of material innovation and clinical feasibility. These limitations must be addressed to ensure successful clinical translation.

1. Material and Structural Constraints. Present biomaterials cannot yet balance mechanics and degradation: fast resorption removes early support, whereas slow breakdown hampers bone formation and remodelling. High porosity favours vessel and cell ingress yet drastically weakens scaffold strength; reconciling porosity with integrity is still difficult. Current printers struggle to recapitulate bone’s multilevel micro-architecture and vascular channels; accuracy and reproducibility for gradient and multi-scale designs must improve.
2. Biological and Functional Limitations. Most scaffolds rely on coatings or factor delivery to spur angiogenesis and repair, yet uncontrolled release, short duration or inactivation prevent lasting, stable vascular integration. Emerging materials—nanoparticles, shape-memory polymers—look promising, yet their long-term biocompatibility and immune or chronic-inflammation risks remain unclear. Existing osteo-inductive and angiogenic cues lack durability and efficiency, falling short of the staged, coordinated regeneration demanded by complex defects.
3. Surgical and Translational Challenges. Combining printed scaffolds with pedicled flaps improves repair but raises operative complexity, demanding advanced microsurgery, imaging and specialised gear—limiting routine use. Patient variability—defect type, vasculature, immunity—hampers protocol standardisation. Most studies remain in small-animal models; lack of large-animal and multicentre trials restrains regulation and evidence-based rollout.
4. Regulatory and Economic Barriers. High-end multi-material printing and co-deposition improve scaffolds, yet lack unified, ISO/GMP-compliant manufacturing and QC standards. Complex post-processing and material costs raise production expenses, hindering scalable, cost-controlled clinical deployment. Composite products—scaffold-drug-flap systems—span multiple biologics, creating convoluted approval routes with no clear regulatory paradigm. Ethical safety, data access and IP policies for smart scaffolds are undeveloped, further hampering their precision-medicine translation.

High-end multi-material printing and co-deposition improve scaffolds, yet lack unified, ISO/GMP-compliant manufacturing and QC standards. Complex post-processing and material costs raise production expenses, hindering scalable, cost-controlled clinical deployment. Composite products—scaffold-drug-flap systems—span multiple biologics, creating convoluted approval routes with no clear regulatory paradigm. Ethical safety, data access and IP policies for smart scaffolds are undeveloped, further hampering their precision-medicine translation.

## 9 Future prospective

### 9.1 Material innovation and cost optimisation

To address the mismatch between degradation and osteogenesis in polymer-ceramic hybrids—and the trade-off between strength and porosity—future work should develop gradient, functionally hybrid materials: employ a rapidly resorbing outer layer (e.g., PLGA) to foster early vascularisation, while an inner core of high-strength, bone-matched degradation secures long-term support. AI-driven topology optimisation could simultaneously cut material usage, and multi-nozzle co-deposition would lower fabrication costs. In addition, incorporating 4-D printing and shape-memory polymers (SMPs) would allow intra-operative, temperature- or pH-triggered shape adaptation, markedly improving implantation accuracy and reducing surgical complexity.

### 9.2 Precision vascularisation strategies

To overcome insufficient vascular density and microcirculatory failure within scaffolds, a microfluidic-assisted dual-channel printing approach could be adopted: ∼200 μm main channels would link directly with host vessels, while 10–50 μm branches deliver ECs, osteoblasts and pro-angiogenic cues, forming a multi-scale network. Coupling this with robot-assisted, nerve-guided flap harvest using intra-operative fluorescence to map nerves and piezo micro-forceps for precise vessel–nerve separation, will preserve neural integrity and provide a stable neuro-regulatory milieu.

### 9.3 Functional and biomimetic design

Present antibacterial coatings are short-lived and osteointegration is slow; a two-stage spatiotemporal delivery system could be engineered: outer antibacterial nanoparticles for rapid microbe suppression, inner bio-ceramic for sustained osteogenesis—achieving osteogenic-antibacterial synergy. Structurally, multi-tier trabecular-mimetic porosity should match cancellous-bone modulus, mitigating stress shielding. Smart responsive materials (pH/temperature-sensitive polymers) can sense the post-operative milieu and release therapeutics, thereby enhancing personalised therapy.

### 9.4 Standardisation, clinical translation and cross-disciplinary synergy

Future work should establish ISO-compliant smart-manufacturing workflows to ensure batch-to-batch consistency and safety, and multicentre trials with large cohorts should verify long-term load-bearing performance. Modular scaffold systems and automated post-processing will lower entry barriers and costs. Concurrently, AI-driven finite-element models should enable patient-specific scaffold design. Interdisciplinarily, a “biomaterialist-clinician-smart-engineer” consortium is required to accelerate translation. Further exploration of intra-operative *in-situ* bioprinting and 4-D programmable therapies and creation of ethical–policy frameworks covering IP, data security and equitable access will strategically support bone-regeneration therapy in precision-medicine settings.

## 10 Conclusion

Research on the transplantation of 3D-printed vascularized artificial bone scaffolds for bone defect repair involves a variety of materials, innovative techniques, and diverse tissue flaps. Biomimetic design approaches can reproduce the structure of natural bone tissue, yielding lighter scaffolds that meet biomechanical needs and thus enhance patient comfort and mobility. Moreover, by integrating smart materials and bioprinting technology, scaffolds can be dynamically tuned according to the patient’s physiological status and external environment, thereby further improving therapeutic outcomes and the patient’s quality of life. Effective vascularization is pivotal to the scaffold’s success in inducing bone tissue regeneration. The integration of 3D-printed, tissue engineering materials, and microsurgical techniques has propelled the development of multifunctional scaffolds and accelerated their clinical translation. This approach has become an emerging technique that provides precise, efficient, and patient-specific construction of vascularized bone tissue engineering scaffolds. This review systematically summarizes the latest developments in the material research and development of 3D-printed artificial bone scaffolds, strategies for loading bioactive substances, and the combination of vascularized tissue flap transplantation for bone defect repair. It covers multiple aspects, including the application of scaffold materials for tissue repair and regeneration, cytokine loading technologies, scaffold design optimization, and the induction of bone vascular regeneration via pedicled tissue flaps. Although 3D-printed technology can precisely fabricate patient-specific artificial bone scaffolds based on individual skeletal anatomy and biomechanical properties, inadequate blood supply may still lead to bone healing failure. Therefore, thorough consideration of safety and regulatory standards is essential before promoting the clinical translation of vascularization strategies. Additionally, tissue flaps, being a highly vascularized covering tissue, play a crucial role in supplying blood and nutrients and in promoting tissue repair. However, because tissue flaps are also rich in nerves, their harvesting process may lead to nerve damage, demanding more advanced microsurgical skills from surgeons. Future research must focus on deep integration of materials and regenerative technologies. Through functionally graded hybrid designs and microfluidic dual-channel vascularization strategies, scaffolds can achieve precision matching of biomechanical-biological properties to patient-specific needs. This exploration will drive convergence across biomedical engineering, smart materials, and computer-enhanced microsurgery, accelerating clinical translation of vascularized 3D-printed bone scaffolds for load-bearing defect repair–ultimately enabling true functional reconstruction of critical-sized bone defects.
